# A Review of the Fungi That Degrade Plastic

**DOI:** 10.3390/jof8080772

**Published:** 2022-07-25

**Authors:** Anusha H. Ekanayaka, Saowaluck Tibpromma, Donqin Dai, Ruifang Xu, Nakarin Suwannarach, Steven L. Stephenson, Chengjiao Dao, Samantha C. Karunarathna

**Affiliations:** 1 Center for Yunnan Plateau Biological Resources Protection and Utilization, Yunnan Engineering Research Center of Fruit Wine, College of Biological Resource and Food Engineering, Qujing Normal University, Qujing 655011, China; hasinie88@gmail.com (A.H.E.); cicidaidongqin@gmail.com (D.D.); 6371105506@lamduan.mfu.ac.th (R.X.); 2 Department of Botany, Faculty of Applied Sciences, University of Sri Jayewardenepura, Gangodawila, Nugegoda 10250, Sri Lanka; 3 Department of Biology, Faculty of Science, Chiang Mai University, Chiang Mai 50200, Thailand; suwan.462@gmail.com; 4 Center of Microbial Diversity and Sustainable Utilization, Chiang Mai University, Chiang Mai 50200, Thailand; 5 Department of Biological Sciences, University of Arkansas, Fayetteville, AR 72701, USA; slsteph@uark.edu; 6 College of Resources and Environment, Yunnan Agricultural University, Kunming 650201, China; chengjiaodcj@163.com

**Keywords:** fungi, global plastic production, plastic waste accumulation, synthetic polymers, multi-gene phylogeny

## Abstract

Plastic has become established over the world as an essential basic need for our daily life. Current global plastic production exceeds 300 million tons annually. Plastics have many characteristics such as low production costs, inertness, relatively low weight, and durability. The primary disadvantage of plastics is their extremely slow natural degradation. The latter results in an accumulation of plastic waste in nature. The amount of plastic waste as of 2015 was 6300 million tons worldwide, and 79% of this was placed in landfills or left in the natural environment. Moreover, recent estimates report that 12,000 million tons of plastic waste will have been accumulated on the earth by 2050. Therefore, it is necessary to develop an effective plastic biodegradation process to accelerate the natural degradation rate of plastics. More than 400 microbes have been identified as capable of plastic degradation. This is the first paper of the series on plastic-degrading fungi. This paper provides a summary of the current global production of plastic and plastic waste accumulation in nature. A list is given of all the plastic-degrading fungi recorded thus far, based on the available literature, and comments are made relating to the major fungal groups. In addition, the phylogenetic relationships of plastic-degrading fungi were analyzed using a combined ITS, LSU, SSU, TEF, RPB1, and RPB2 dataset consisting of 395 strains. Our results confirm that plastic-degrading fungi are found in eleven classes in the fungal phyla Ascomycota (Dothideomycetes, Eurotiomycetes, Leotiomycetes, Saccharomycetes, and Sordariomycetes), Basidiomycota (Agaricomycetes, Microbotryomycetes, Tremellomycetes, Tritirachiomycetes, and Ustilaginomy-cetes), and Mucoromycota (Mucoromycetes). The taxonomic placement of plastic-degrading fungal taxa is briefly discussed. The Eurotiomycetes include the largest number of plastic degraders in the kingdom Fungi. The results presented herein are expected to influence the direction of future research on similar topics in order to find effective plastic-degrading fungi that can eliminate plastic wastes. The next publication of the series on plastic-degrading fungi will be focused on major metabolites, degradation pathways, and enzyme production in plastic degradation by fungi.

## 1. Introduction

Plastic is one of the most abundant human-produced, versatile materials on the earth. Its high stability and long durability facilitate its integral role in our day-to-day lives, from the kitchen to an industrial level [1,2]. Plastic is a polymer and consists of the elements carbon, hydrogen, silicon, oxygen, chlorine, and nitrogen [1]. Plastic production can be bio-based or synthetic. Bio-based plastics are made from natural compounds such as lignin, cellulose, hemicellulose, terpenes, vegetable oils, carbohydrates, and food waste [3,4,5]. In contrast, crude oil is the main component of synthetic plastics [6,7], which are generally referred to as non-biodegradable [8].

The present study is focused on synthetic plastics. Seven major types of synthetic plastic are used around the world. These are polyethylene terephthalate (PET), high-density polyethylene (HDPE), polyvinyl chloride (PVC), low-density polyethylene (LDPE), polypropylene (PP), polystyrene (PS), and various other plastics that include acrylic, polycarbonates, polylactic acid (PLA), fibers, and nylon [3,9].

Global plastic production is rising year by year. In 2018, the annual global plastic production was 359 million tons, while it was 368 million tons in 2019 [10]. China is the world leader in plastic production, accounting for 31% of world plastic production in 2019 [10]. Although the production of new plastic increases annually, the rate of plastic waste management and plastic recycling rate has not reached precise levels [10]. In Europe, total plastic production in 2018 was 61.8 million tons. However, the amount of plastic post-consumer waste collected in Europe in 2018 was only 9.4 million tons [10]. The estimated global total for virgin plastic production was 8300 million tons in 2017 [11]. The amount of plastic waste accumulated as of 2015 was 6300 million tons; from that, only 9% were recycled, 12% were incinerated, and 79% were placed in landfills or introduced to the natural environment [11]. Every year 25 million tons of synthetic plastics are accumulated along seacoasts and in terrestrial environments [12]. In 2019, the amount of plastic waste released into the aquatic environments was 6.1 million tonnes (Mt), and 1.7 Mt flowed into oceans. Currently, there has been a dramatic increase. At least eight million tons of plastic get into oceans, which have the potential of breaking down into tiny microplastics that possibly make their way into our food chains and result in unknown effects [13]. Current plastic accumulation in reverse is 109 Mt [14]. The build-up of plastics in rivers implies that leakage into the ocean will continue for decades, even if mismanaged plastic waste could be significantly reduced [14]. Current estimations reveal that 80% of marine litter accumulation is due to plastic debris [4]. This marine plastic waste accumulation in amounts is around 30 Mt. The total amount of plastic waste only in the ocean is expected to grow from 50 million tons in 2015 to 150 million tons by 2025 [15]. If current production and waste management trends continue, roughly 12,000 million tons of plastic waste will be in landfills or the natural environment by 2050, with an annual accumulation of ~339 million tons [11,16].

Moreover, when considering the plastic accumulation based on major plastic producing regions globally, in just Europe, 75,000 to 300,000 tons of microplastics accumulate every year [4]. In the United States, the landfill rate for discarded plastics exceeds 75% [17]. 

Therefore, these various types of plastics, after being used and then discarded, not only pervade in every nook and cranny of the planet but also cause major negative impacts at the ecosystem level [18]. At present, plastic pollution has become one of the major global environmental issues [4], and it is the leading cause of biodiversity reduction. Polyethylene causes blockages in the intestines of birds, fish, and marine mammals. Entanglement in or ingesting this waste has endangered hundreds of different species [19]. Fragmented plastic debris, referred to as microplastics, were reported entrapping in subtropical gyres that run over a million square kilometers. These microplastics ingested by a wide range of organisms subsequently cause substantial negative impacts on their existence [20]. Moreover, plastics can cause health effects; for instance, airborne microplastics can be inhaled, thus representing a direct risk to human health [21].

The natural degradation rate of plastic is extremely slow, and that causes plastics wastes to accumulate in all components of the environment [22,23]. The long chain polymer structure, high molecular weight, and hydrophobicity cause plastics to be resistant to biodegradation [24]. In fact, some plastics take up to 1000 years to degrade [25]. These facts are the reason for the rapid plastic accumulation in natural environments. Hence, developing an efficient process to accelerate the plastic degradation rate is essential to avoid this annual accumulation. Several solutions have been provided in the scientific community and have been experimentally proved to some extent. Those methods include photo-degradation (degraded by light), chemical degradation, thermal degradation (degraded by heat), irradiation using gamma rays, and biodegradation (degraded by biological additives or microorganisms) [25,26,27]. However, a method with minimum harmful effects to nature but without or at most minimum toxic by-products is required [27]. The processes such as photo-degradation, chemical degradation, thermal degradation, and irradiation using gamma rays cause many negative impacts on nature, such as accumulation of heavy metals in ecosystems and disturbance in natural ecosystem functioning. Moreover, those methods require high costs and energy levels to perform. Therefore, scientists across the globe have tended to investigate better biodegradation methods that do not result in harmful effects and represent an eco-friendly approach for managing plastics [27]. Furthermore, biodegradation is a proper solution since it is cost-effective and does not require much energy. More than 400 microbes have been recorded to be capable of plastic degradation [28]. The present study was focused only on plastic-degrading fungi among plastic-degrading microbes.

Several studies have been carried out on plastic-degrading fungi. For example, Lacerda et al. [29] investigated the fungi from the plastisphere within the aquatic environments of the western South Atlantic and Antarctic Peninsula. Microorganisms and enzymes that are able to degrade a variety of generally used synthetic plastics were comprehensively summarized. The microbial metabolic pathways for plastic depolymerization products and the current attempts toward utilization of such products as feedstocks for microbial production of chemicals with high value were highlighted by Ru et al. [30]. Sánchez [31] described the natural and unique ability of fungi to invade macro- and microplastic substrates by using enzymes that have the capacity to detoxify pollutants. Sáenz et al. [32] experimented with biodegradation of low-density polyethylene (LDPE) by *Aspergillus niger* and *A*. *terreus* to increase the degradation rate without any co-substrate or photothermal treatment. Iram et al. [33] reviewed some of the most common strategies for the degradation of various types of polymers, along with a list of potential microbes capable of feeding on them. The role of marine fungi was also recently reviewed by Zeghal et al. [34]. Microbial degradation of plastics was reviewed by Kale et al. [22]. However, a recent complete study on plastic-degrading fungi in all environments, including both aquatic and terrestrial environments, which addresses their detailed phylogenetic relationships, is lacking. As such, the information presented herein reviews plastic-degrading fungi reported thus far and their phylogenetic relationships.

The present study attempted to summarize current plastic production and plastic waste accumulation in natural environments worldwide. Moreover, a list of all taxa of fungi known to be capable of degrading plastic-degrading fungi was developed from the available literature, and notes were compiled on the major fungal groups involved. In addition, the phylogenetic relationships of plastic-degrading fungi were analyzed based on multi-gene analyses (ITS, LSU, SSU, TEF, RPB1, and RPB2). As such, the overall objective of the study was to summarize all currently known basic information on plastic-degrading fungi to influence future research on similar topics.

## 2. Materials and Methods

### 2.1. Literature Review

The data presented herein was acquired from several different sources—the published literature, online databases, and personal communication. We selected seven major synthetic plastic types used around the world based on recent literature [3,9] and the species of fungi reported to degrade those plastic types. Plastic-degrading fungal species data were collected primarily from “Google Scholar”, “Research Gate”, “PubMed”, and “Web of Science”. The main phrases used in the online literature search for the plastic types mentioned above and the taxa of fungi involved in their biodegradation were “plastic types”, “global plastic production”, “global plastic waste management”, “fungal bio-degradation of plastics”, and “bio-degradable plastics”. A list of major types of synthetic plastic with their major uses and annual production was prepared (Table 1). Taxa of fungi reported to degrade plastics are listed in Table 2.

### 2.2. Phylogenetic Analyses

#### 2.2.1. Taxon Sampling

A data matrix containing 395 taxa, including four out-group taxa (*Basidiobolus ranarum* AFTOL-ID 301, *Basidiobolus ranarum* ARSEF 260, *Basidiobolus ranarum* ATCC 14449, and *Olpidium brassicae* AFTOL-ID 633) were generated in the present study. ITS, LSU, SSU, TEF, RPB1, and RPB2 sequence data and their GenBank accession numbers are provided in Table S1.

#### 2.2.2. Initial Phylogenetic Analyses

**Figure 1:**
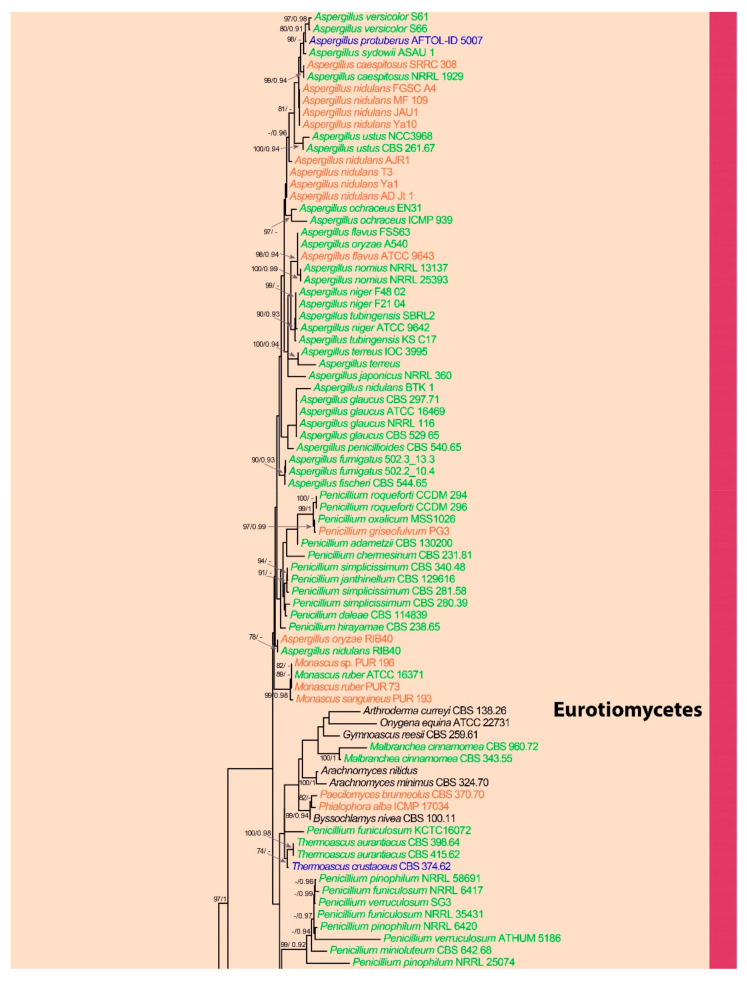

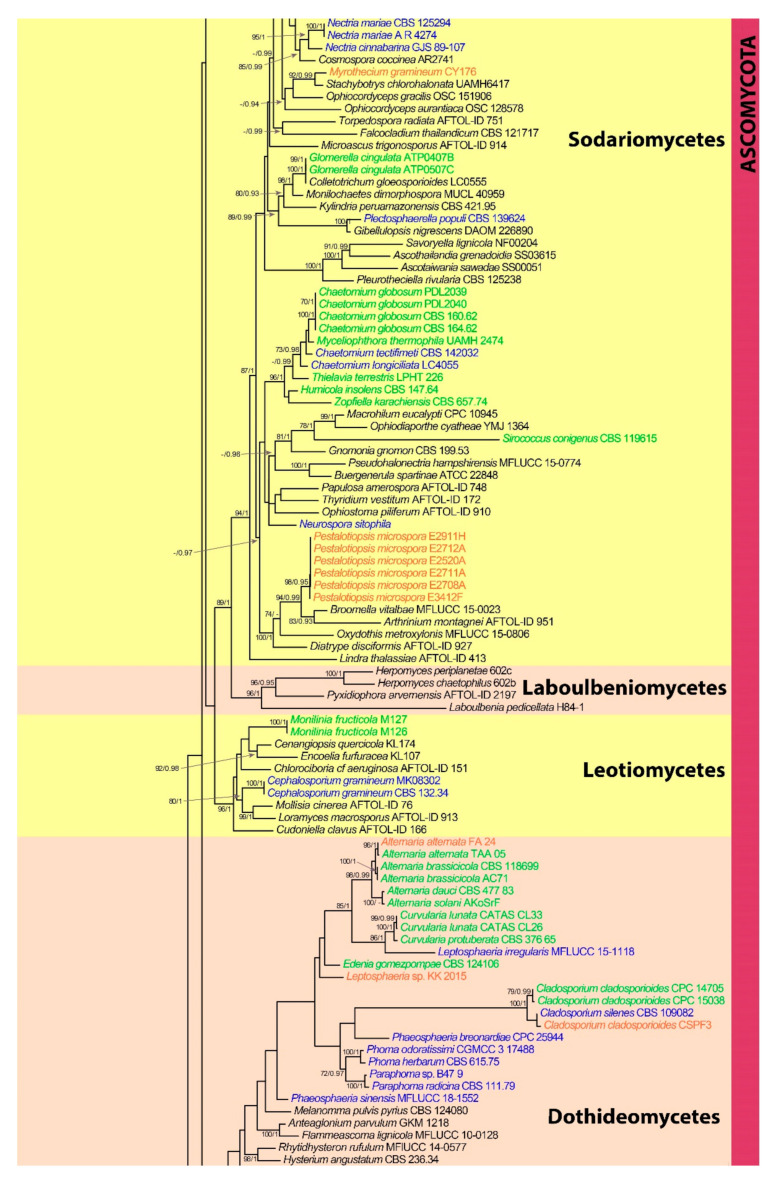

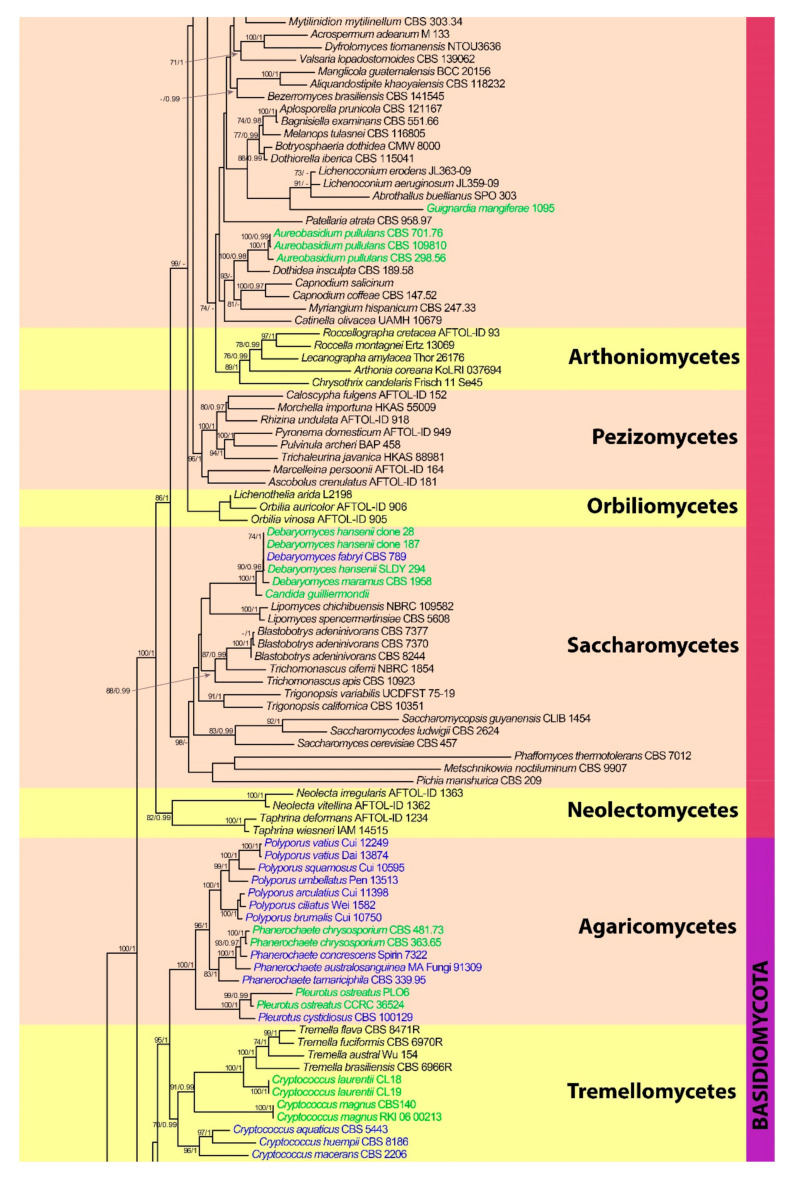

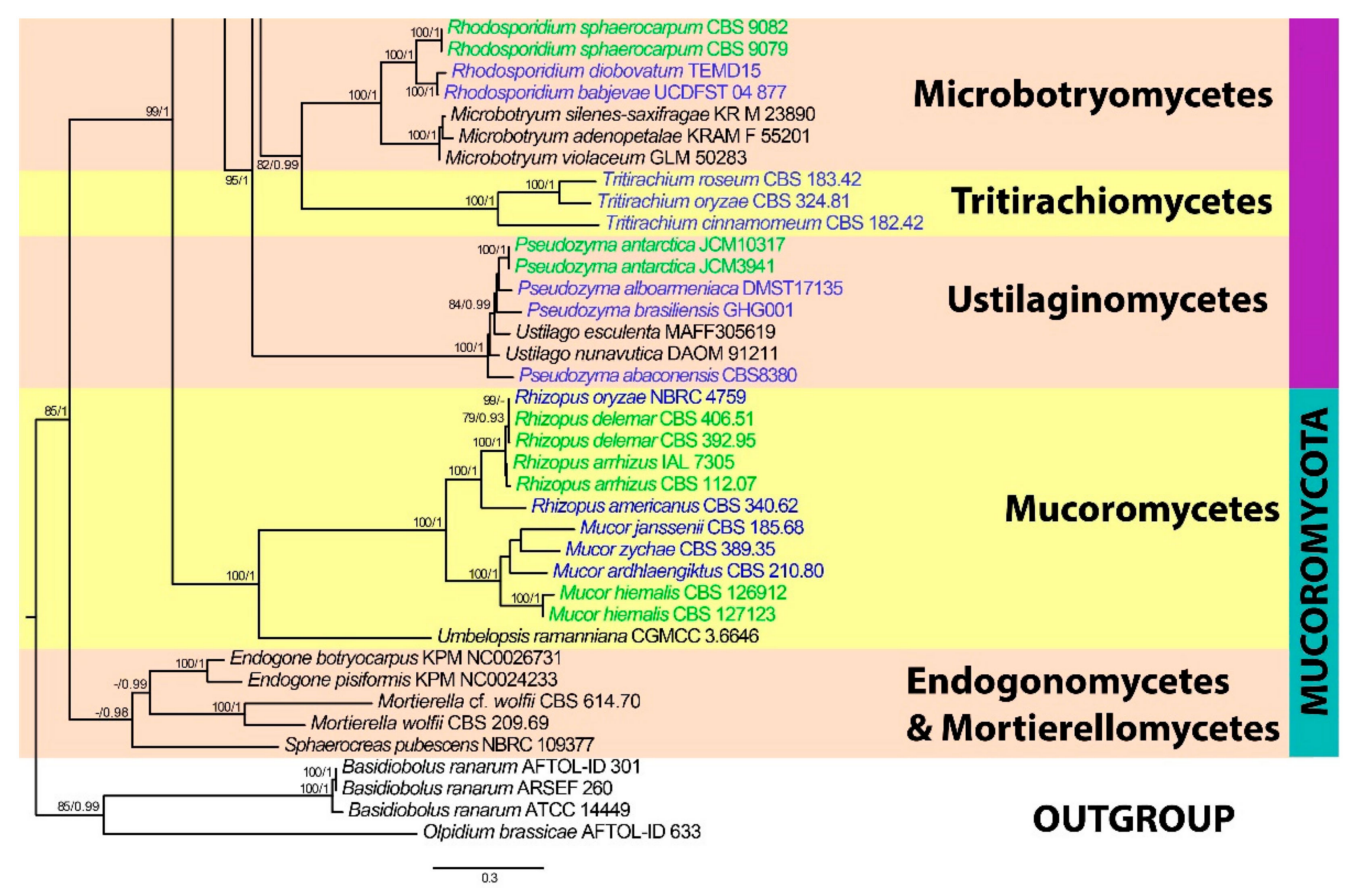
Phylogenetic relationships of plastic-degrading fungi. Phylogram generated from a maximum likelihood analysis of ITS, LSU, SSU, TEF, RPB1 and RPB2 fungal sequence data. MLBP values ≥ 70% and BYPP ≥ 0.90 values are given as the first and the second set of numbers near the nodes. Strain/culture numbers are given after the taxon names. The tree is rooted with *Basidiobolus ranarum* AFTOL-ID 301, *Basidiobolus ranarum* ARSEF 260, *Basidiobolus ranarum* ATCC 14449, and *Olpidium brassicae* AFTOL-ID 633. Strains in Red: Strains directly reported as plastic degraders. Green: Other strain of the same species reported as plastic degraders. Blue: Other species in the same genus reported as plastic degraders.

**Figure 2:**
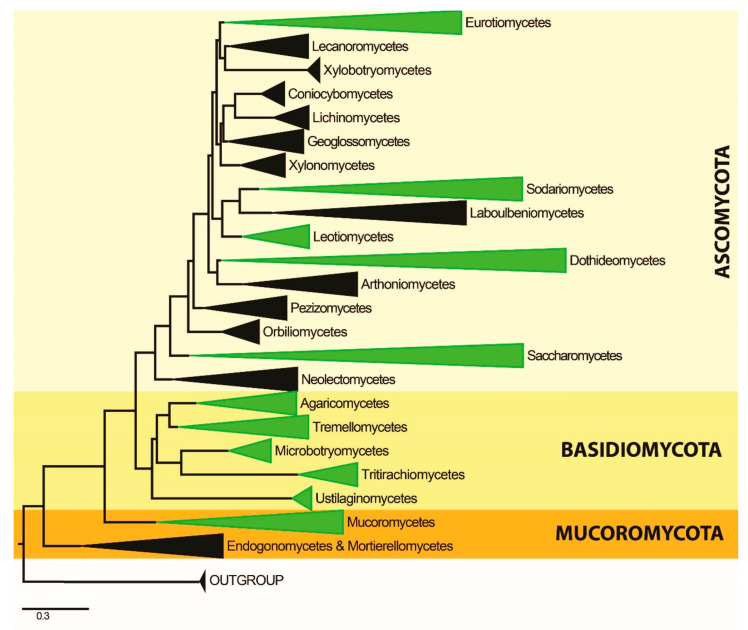
Compressed overview of the phylogram for the phylogenetic relationships of plastic-degrading fungi. Class level taxonomic ranks in Green include fungal species that were reported as plastic degraders.

Multiple sequence alignments for each gene (ITS, LSU, SSU, TEF, RPB1, and RPB2) were generated with MAFFT version 7 (http://mafft.cbrc.jp/alignment/server/, accessed on 22 April 2022). Long alignment gaps were removed using trimAl [123] available at Phylemon2 web server [124] and manually adjusted in BioEdit v. 7.0.4 [125] where necessary. The individual datasets were concatenated into a combined dataset using FaBox (1.41) [126]. Ambiguously aligned regions were excluded, and gaps were treated as missing data. Maximum likelihood phylogenetic analyses were performed in the CIPRES web portal [127] using RAxML-HPC2 on XSEDE (8.2.12) tool [128]. The bootstrap analysis for each ML tree was performed with 1000 thorough bootstrap replicates with the same parameter settings using the GTR+G+I substitution model selected by MrModel Test 2.2 [129]. Posterior probabilities (PP) [130,131] were determined by Markov Chain Monte Carlo sampling (MCMC) in MrBayes v. 3.0b4 [132]. Four simultaneous Markov chains were run for 20,000,000 generations, and the trees were sampled every 1000th generation. MCMC heated chain was set with a “temperature” value of 0.2. The distribution of loglikelihood scores was examined to determine the stationary phase for each search and to decide if extra runs were required to achieve convergence using the program Tracer 1.5 [133]. All sampled topologies beneath the asymptote (40%) were discarded as part of a burn-in procedure, while the remaining trees (12,000) were used for calculating posterior probabilities in the majority rule consensus tree. Bayesian Posterior Probabilities (BYPP) equal to or greater than 0.90 are given below or above each node (Figure 1). The resulting trees were viewed with FigTree v.1.4.0 [134]. The compressed overview of the phylogram resulting from the phylogenetic analysis is presented in Figure 2, where classes including plastic-degrading fungi are colored green.

## 3. Results

### 3.1. Phylogenetic Analyses

The present study investigated the phylogenetic relationships of plastic-degrading fungi based on an analysis of ITS, LSU, SSU, TEF, RPB1, and RPB2 sequence data. The alignment of combined genes included 6289 bp (ITS-1–388, LSU-389–1434, SSU-1435–2502, TEF-2503–3382, RPB1-3383–4569, and RPB2-4570–6289). The topology of the tree from maximum likelihood analysis was similar to the tree from Bayesian analysis. The best scoring RAxML tree with a final likelihood value of −275,048.218811 is presented. The matrix had 5830 distinct alignment patterns, with 67.51% undetermined characters or gaps.

The phylogenetic tree (Figure 1 and Figure 2) representing those taxa of fungi known to be able to degrade fungi comprises three major clades, with these representing the three major phyla Ascomycota, Basidiomycota, and Mucoromycota. The basal clade Mucoromycota includes the class Mucoromycetes. Consequently, these taxa are the basal groups of plastic degraders in the fungi kingdom. The phylum Basidiomycota also can be recognized as a clade, and it is comprised of the Agaricomycetes, Microbotryomycetes, Tremellomycetes, Tritirachiomycetes, and Ustilaginomycetes. For Clade 3, the upper clade is the phylum Ascomycota and includes the Dothideomycetes, Eurotiomycetes, Leotiomycetes, Saccharomycetes, and Sordariomycetes. Based on the phylogeny of the present study, the Eurotiomycetes are the most evolved group of plastic-degrading fungi. The highest numbers of plastic-degrading fungi are included in Clade 3. 

### 3.2. Phylum Ascomycota

#### 3.2.1. Class Dothideomycetes

The Dothideomycetes is the largest class within the phylum Ascomycota. Most of the taxa within this class are recorded as saprobes in various habitats and substrates [135]. Our investigations revealed that some members of Dothideomycetes are capable of plastic degradation. Most of the recorded species of the Dothideomycetes belong to the order Pleosporales, but a few taxa are recorded from the Dothideales and Botryosphaeriales (Table 2, Figure 1 and Figure 2). Plastic degraders in the Dothideomycetes have the ability to degrade LDPE, PUR, PS, PCL, PEA, PPA, PBA, HDPE, PVC, PE, PU, and Sky-Green plastics (Table 2). Recent studies on plastic-degrading members of the Dothideomycetes are those of Khruengsai et al. [64] and Brunner et al. [6]. In the phylogenetic tree, the Dothideomycetes formed a well-supported clade sister to the Arthoniomycetes. Most of the plastic-degrading Dothideomycetes were placed in the upper subclade of the main Dothideomycetes clade, and the rest were grouped in a basal subclade (Figure 1 and Figure 2).

#### 3.2.2. Class Eurotiomycetes

The Eurotiomycetes are extremely common saprobes in diverse habitats and substrates [136]. Based on our results, most plastic-degrading fungal records belong to the Eurotiomycetes. Many plastic-degrading members of the Eurotiomycetes are taxonomically placed under the Eurotiales, and the most common plastic-degrading fungal genera are *Aspergillus* and *Penicillium* (Table 2, Figure 1 and Figure 2). The plastic types they are reported to degrade are HDPE, LDPE, PCL, PE, PVC, PS-PUR, PEA, PPA, PBA, PHB, Poly[3HB-co-(10 mol%) 3HV], Sky-Green, PHV, PBS, PLA and PVC (Table 2). Recent studies on plastic-degrading Eurotiomycetes are those of Rani & Singh [12], Ndahebwa Muhonja et al. [54], Bermúdez-García et al. [57], Laila [63], Khruengsai et al. [64], Munir et al. [65], Alshehrei [69], Sangale et al. [2], Duan et al. [90], El-Morsy et al. [92], Brunner et al. [6] and Ojha et al. [26]. The Eurotiomycetes is the uppermost clade within our phylogram (Figure 1 and Figure 2). The genera *Aspergillus* and *Penicillium* contain a large number of species with a worldwide distribution and a huge range of ecological habitats [137]. They are mostly widespread saprobes and can be found in both indoor and outdoor environments, including in both the air and soil. In addition, some species of *Aspergillus* and *Penicillium* have the ability to grow under extreme conditions [137]. Hence, further research on these genera would provide better solutions for the environmental accumulation of plastics.

#### 3.2.3. Class Leotiomycetes

Most of the Leotiomycetes are saprobes on a wide variety of substrates. However, this class also includes many important plant pathogens [138]. Our results showed that the few records of plastic-degrading fungi are phylogenetically related to the Leotiomycetes (Table 2, Figure 1 and Figure 2). In the phylogenetic tree of this study, the Leotiomycetes formed a well-supported clade sister to the Laboulbeniomycetes (Figure 1 and Figure 2). However, there is a single record found that clearly belongs to the Leotiomycetes and possibly has the ability of plastic degradation [92]. The other records that grouped within Leotiomycetes in our phylogeny were *Cephalosporium gramineum*, which is currently placed under the Sordariomycetes. Therefore, studies with a wide range of taxon sampling are required to resolve the phylogenetic position of *Cephalosporium gramineum*. Furthermore, a recent study on the biodegradation of bio-based and biodegradable plastic, polybutylene succinate-co-adipate (PBSA), identified a species (*Tetracladium furcatum)* within the Leotiomycetes that has the ability to degrade PBSA [139]. However, recent studies on synthetic plastic-degrading members of the Leotiomycetes are very few. As a result, additional studies on the Leotiomycetes are required to assess their ability to degrade plastics.

#### 3.2.4. Class Saccharomycetes

The Saccharomycetes is a small class of yeasts with a single order of about 1000 known species, which are classified under the Ascomycota [140]. Most of the Saccharomycetes are saprobes, and few are recorded as human and plant pathogens [140]. The present study discovered five records of five members of the Saccharomycetes capable of degrading plastics (Table 2, Figure 1 and Figure 2). The Saccharomycetes is the second basal clade within the phylum Ascomycota. All Saccharomycetes members that are plastic degraders are grouped in the upper subclade of the main Saccharomycetes clade (Figure 1 and Figure 2). *Arxula*, *Candida,* and *Debaryomyces* are the genera to which those plastic-degrading members of the Saccharomycetes belong [43,71]. Some members of the Saccharomycetes are widely used in industrial and biotechnological processes. Species such as *Saccharomyces cerevisiae* are model organisms in many types of research [140]. A recent study used Genetic engineering techniques on two strains of *Saccharomyces cerevisiae* to produce the heterologous protein Polyethylene Terephthalate (PET) hydrolase enzyme, which has been shown to have the capability of degrading PET into its subsequent monomers [141]. We believe future research on the Saccharomycetes would find a better solution for plastic accumulation in nature.

#### 3.2.5. Class Sordariomycetes

The Sordariomycetes is the second largest class within the phylum Ascomycota. The majority of the Sordariomycetes are saprobes, but the group also includes some important plant pathogens [142]. They have a wide ecological distribution in both terrestrial and aquatic habitats [142]. Our research recorded many members of the Sordariomycetes with the ability to degrade plastics (Table 2, Figure 1 and Figure 2). The plastic types they are reported to degrade are PE, PS, PHB, Poly[3HB-co-(10 mol%) 3HV], PUR, PS-PUR, HDPE, LDPE, PVC, PCL, PEA, PPA, and PBA (Table 2). Recent studies of plastic-degrading Sordariomycetes are those of Munir et al. [65], (Yang et al. [93], Brunner et al. [6], and Khruengsai et al. [64]. The Sordariomycetes formed a middle clade within the main Ascomycota clade that is sister to the Laboulbeniomycetes (Figure 1 and Figure 2). Many plastic degraders in the Sordariomycetes are classified under the Hypocreales, while others belong to the Amphisphaeriales, Glomerellales, Phyllachorales, and Sordariales. Even though the Eurotiomycetes include the highest number of records of plastic-degrading fungi, the Sordariomycetes contain the highest number of genera with the capability to degrade plastics.

### 3.3. Phylum Basidiomycota

#### 3.3.1. Class Agaricomycetes

The Agaricomycetes is a morphologically diverse class of macrofungi within the Basidiomycota, containing around 36,000 described species. They are ecologically diverse and include saprobes, mycorrhizal symbionts, and pathogens [143]. Moreover, the Agaricomycetes encompasses several important commercially growing edible mushrooms [144]. The present study found several records of Agaricomycetes with the ability to degrade plastics (Table 2, Figure 1 and Figure 2). They are reported to degrade Poly[3HB-co-(7 mol%) 3HV], LDPE, PVC, polyethylene, and PHB (Table 2). Additionally, da Luz et al. [16] performed a study on plastic-degrading Agaricomycetes. They investigated the degradation of oxo-biodegradable plastic bags and green polyethylene by *Pleurotus ostreatus*. The Agaricomycetes is the upper clade within phylum Basidiomycota, and it is highly statistically supported within the present phylogeny (Figure 1 and Figure 2). Further studies of edible mushrooms belonging to the Agaricomycetes and their ability to degrade plastics would increase world food production and reduce the plastic accumulation in nature.

#### 3.3.2. Class Microbotryomycetes

The Microbotryomycetes include mainly mycoparasites, saprobic yeasts, and plant pathogens [145]. A single record of plastic-degrading fungi was found in the order Sporidiobolales of the Microbotryomycetes in the present study (Table 2, Figure 1 and Figure 2). In our phylogenetic tree, the Microbotryomycetes formed a well-supported clade sister to Tritirachiomycetes (Figure 1 and Figure 2).

#### 3.3.3. Class Tremellomycetes

The Tremellomycetes are classified under the Basidiomycota and consist of saprobic yeasts, dimorphic taxa, and species that form hyphae and/or complex fruiting bodies [146]. The present study identified a few records from the Tremellomycetes that are capable of degrading plastics (Table 2, Figure 1 and Figure 2). All the records belong to the genera *Cryptococcus* and *Papiliotrema* (Tremellales), andthey are capable of degrading PCL, PBS, and PBSA (Table 2). The Tremellomycetes formed a well-supported clade sister to Agaricomycetes in the present phylogenetic tree (Figure 1 and Figure 2). A recent study on synthetic plastic-degrading Tremellomycetes was published by Hung et al. [114]. A recent study on biodegradable plastic mulch films (BDMs) and their associated soil microbial communities found that the Tremellomycetes are capable of degrading agriculturally-weathered BDMs [147].

#### 3.3.4. Class Tritirachiomycetes

The Tritirachiomycetes is a small class within the Basidiomycota that is made up of filamentous fungi. They are mainly saprobes, but some have been recorded as human pathogens [148]. A single record of a fungus from this class that is capable of degrading plastics was found in the present study (Table 2, Figure 1 and Figure 2). The Tritirachiomycetes formed a well-supported clade sister to the Microbotryomycetes in our phylogenetic tree (Figure 1 and Figure 2).

#### 3.3.5. Class Ustilaginomycetes

The majority of the Ustilaginomycetes are economically important plant pathogens. They are usually unicellular yeasts (sporidia), but some are simple multicellular forms, such as a pseudomycelium, multicellular cluster, or mycelium [149]. Moreover, the Ustilaginomycetes have a comparatively short life cycle, which makes them easy to handle under laboratory conditions. As a result, the Ustilaginomycetes can be considered model organisms for studying fungi [149]. The present study found a single record from the Ustilaginomycetes of a fungus capable of degrading plastics (Table 2, Figure 1 and Figure 2). The Ustilaginomycetes is the basal clade within the main Basidiomycota clade, and it is statistically highly supported (Figure 1 and Figure 2).

### 3.4. Phylum Mucoromycota

Class Mucoromycetes

The Mucoromycetes are a class within the phylum Mucoromycota and consist of mainly filamentous fungi with a saprobic lifestyle. Several species are also life-threatening human pathogens, plant parasites, and food spoilage organisms. Moreover, members of the Mucoromycetes are used as a traditional fermenting agent for Asian and African foods, such as soybean products and several varieties of European cheese. Fungi belonging to the Mucoromycetes are common in the environment and able to colonize all kinds of wet, organic substrates [150]. The present study found several Mucoromycetes fungi with the ability to degrade plastics. All the recorded plastic-degrading members of the Mucoromycetes belong to the genera *Mucor* and *Rhizopus*. These fungi are capable of degrading PHB, HDPE, LDPE, PVC, PCL, polyalkylene dicarboxylic acids, PPA, and PET copolymers with dicarboxylic acids (Table 2). A recent study of plastic-degrading Mucoromycetes was that of Pardo-Rodríguez & Zorro-Mateus [106]. The Mucoromycota formed the basal clade within our phylogenetic tree (Figure 1 and Figure 2). However, the phylum presented a polyphyletic nature in the present phylogenetic tree and was separated into two basal clades.

## 4. Discussion

As global plastic production and plastic waste accumulation increase rapidly, we need to look for a quick and efficient solution to save nature and, indeed, the entire planet [4,7]. The problem becomes even more acute as the natural degradation rate of all types of plastic is very slow [23]. Hence, it is essential to look at ways to accelerate plastic degradation methods. Several solutions that have been proposed are photo-degradation (degrade by light), chemical degradation, thermal degradation (degrade by heat), irradiation using gamma rays, and biodegradation (degrade by biological additives or microorganisms) [25,26,27]. Biodegradation has been suggested as the best solution as it is an eco-friendly approach [27]. However, based on the results obtained in previous studies, people select biodegradation since the other options are not cost-effective [26]. In this study, we reviewed the literature on plastic-degrading fungi from different sources and summarized a list of records on plastic degraders in the fungi kingdom. Moreover, we analyzed phylogenetic relationships of plastic-degrading fungi based on a combined ITS, LSU, SSU, TEF, RPB1, and RPB2 dataset from 395 strains and provided brief taxonomy at the level of class.

The present study identified more than 200 records of fungi capable of degrading different plastic types under a wide range of conditions. These are listed in Table 2. Most plastic-degrading fungal records are found in the class Eurotiomycetes of the phylum Ascomycota. However, a wide range of generic level diversity in plastic-degrading fungi was found in the class Sordariomycetes of the phylum Ascomycota. Considering phylum level diversity in relation to the ability to degrade plastic, most of the plastic degraders belong to the phylum Ascomycota. The second group with the highest number of recorded plastic degraders was in the phylum Basidiomycota, with a few records also found in the phylum Mucoromycota. However, very few sequences are available directly from the plastic-degrading fungi. Hence, we used sequence data from another strain of the same species, which the species was recorded as a plastic degrader. Furthermore, to establish a detailed and accurate phylogenetic tree for plastic-degrading fungi, sequence data need to be obtained exactly from the strains which were recorded as plastic-degrading fungi. Moreover, future studies on whole-genome sequencing or sequencing of more gene regions and evolutionary relationships analyses of plastic-degrading fungi concerning the specific plastic type the fungi can degrade would be beneficial to develop commercially available plastic degrading fungal strains.

Recent studies have reported some common saprobic fungi and plant pathogenic fungi that can degrade plastic [6]. Our study agrees with this statement as we found many plastic degraders that belong to the Eurotiomycetes and Dothideomycetes when most of the saprobic fungi are taxonomically members of the Sordariomycetes, where many important plant pathogens are classified. Moreover, recent research has reported that fungi that can degrade complex C-polymers, such as lignin and protein, can also degrade plastics [6]. Therefore, future studies of the Dothideomycetes, Eurotiomycetes, and Sordariomycetes are essential. Since these classes include plant pathogens, saprobes, and degraders of complex C-polymers, it is possible that they can degrade plastics. Moreover, these findings also proved that enzymes produced by common saprobes and plant pathogens can degrade or even transform plastics. Hence further studies on the metabolic pathways of those fungi and the discovery of the essential enzymes involved in plastic degradation can significantly contribute to reducing plastic accumulation in nature.

Plastics accumulating in the marine environment are an increasingly important environmental issue [34]. Most of the plastic waste is accumulated in the sea, and there will be more plastics in the sea than fish by 2050. Recently, plastic-fungi interactions in marine environments were reviewed by Zeghal et al. [34]. The same study listed more than 60 records of fungi identified to the species level and belonging to the Ascomycota and Basidiomycota as having the ability to degrade plastic debris in marine environments [34]. Our study listed plastic-degrading fungi from all environmental conditions on the earth. When comparing the number of records, only about twenty-five percent of records are from marine environments, even though the highest environmental pollution from plastics is recorded from marine environments. This clearly shows the requirement for more research on plastic-degrading fungi in marine environments. Moreover, Zeghal et al. [34] concluded that future studies are required to identify species of fungi that can degrade plastics in the marine environment. They also concluded that these studies should address their enzymatic potential, which might then serve biotechnological applications for plastic waste bioremediation [34]. Furthermore, the effect of physicochemical parameters in marine environments (such as salinity and pH) on plastic-degrading fungi and the differences between the metabolic pathways of marine plastic degraders from terrestrial plastic degraders need to be focused on in future research fields to save the sea from plastic accumulation.

Plastic use in the agricultural sector is growing rapidly. Some of the main examples of LDPE use are in greenhouse and mulch film as well as in structures to provide crop protection against environmental conditions and insects and pests. HDPE is used in containers for pesticides and pesticides as well as in containers for nurseries or slips for irrigation, and PP is used in containers for nurseries or floating covers and polystyrene in trays for a plant nursery [151]. Recent research by Khan & Stevenson [151] investigated the plastic-degrading ability of soil fungi and found that *Aspergillus tubingensis* can successfully colonize plastic surfaces. Additionally, they found that the enzymes this fungus produces can break the chemical bonds between the plastic molecules. Moreover, the genus *Aspergillus* is very important in plastic degradation. Several species from different habitats have been reported that degrade various plastic types (Table 2). As such, further research on the members of the genus *Aspergillus* and their ability to degrade plastics needs more attention. Not only *Aspergillus*, but many other fungi have also been reported (Table 2), including the ability to degrade multiple plastic types by the same fungi. Some examples are *Chaetomium globosum* that can degrade HDPE, LDPE, PVC, PCL, PEA, PPA, and PBA, *Fusarium solani* that can degrade LDPE, HDPE, PVC, PCL, PS-PUR, PHB, and PET, and *Penicillium funiculosum* that can degrade PCL, PHB, PHV, Poly[3HB-co-(7, 14%) 4HB], Poly[3HB-co-(7, 27, 45, 71%) 3HV], PEA, PPA, and PBA. Those fungi that can degrade multiple plastics have also been recorded from different habitats. Hence, they are the perfect candidates for strain improvements for commercial applications. Molecular studies of those plastic-degrading fungi, specifically on genes related to the metabolic pathways of those fungi involved in plastic degradation, are critical. Simultaneously, even though there are commercially developed strains for plastic degradation in agricultural lands, there must be experimental confirmation that those fungi do not have pathogenic effects on crop plants and the beneficial flora and fauna associated with the farmlands.

Low-Density Polyethylene (LDPE) is the most common type of plastic, generally known as polythene [9,152]. There are more than 15 species of fungi, including several members of *Aspergillus* recorded as capable of degrading LDPE (i.e., *Aspergillus caespitosus*, *Aspergillus flavus*, *Aspergillus fumigatus*, *Aspergillus nidulans*, *Aspergillus niger*, *Aspergillus nomius*, *Aspergillus ochraceus*, *Aspergillus oryzae*, *Aspergillus terreus*, *Chaetomium globosum*, *Chrysonilia setophila*, *Diaporthe italiana*, *Fusarium solani*, *Gliocladium virens*, *Phoma* sp., *Thyrostroma jaczewskii*, *Trichoderma hamatum*, and *Mucor hiemalis*) (Table 2). Therefore, we suggest that the genus *Aspergillus* would be a perfect fungal genus for developing commercial levels to degrade plastics. For the basement of such a lengthy biotechnological application, comprehensive molecular studies related to the ecology of the genus *Aspergillus* are very important. Moreover, investigations on enzyme production and metabolic pathways of the genus *Aspergillus* are helpful research areas.

The slow degradation rate of synthetic plastics and the growing demand for increasingly more limited oil reserves drive the effort to produce bio-based plastics [3,16]. Bio-based plastics originate from renewable resources such as plant and animal waste products from industry [3]. However, the global markets for bio-based plastics are still small. The annual global production of bio-based plastics in 2020 was approximately 2 Mt [153]. Moreover, bio-based plastics do not represent a better solution for the slow degradation rate of synthetic plastics or plastic accumulation in nature. Recent records indicate that only half of bio-based plastics are biodegradable, while the other half is non-biodegradable [3,4,5]. Bio-based plastics naturally degrade only to some extent. For the remainder, we once again need to look for suitable microorganisms that are capable of their biodegradation.

Although there are more than 400 records of plastic-degrading microbes, including over 200 records of species of plastic-degrading fungi, many studies have failed to differentiate losses caused by the leaching or degradation of polymers [28]. Moreover, many studies have used more highly crystalline polymers with species of fungi under laboratory conditions [28]. Hence, even the species of fungi involved provided few clues as to their ability to degrade those crystallized polymers. There was no confirmation of their biodegradation rate in a natural environment where large-sized plastic wastes have accumulated. Herein we propose a more detailed in situ research approach that delivers a clearer picture of plastic-fungi interactions, including changes in the polymer structure, mass loss, and confirmation of fungal strains and their enzymatic activity with related genes to degrade large-sized plastic polymers. Furthermore, genetic and biotechnology research is required to identify enzymes and their related genes for plastic degradation from currently identified plastic-degrading fungi. We expect to continue our following review on enzyme production and metabolic pathways of plastic-degrading fungi and their evolutionary relationships. This information can then be applied to develop more powerful hybrid fungal species to degrade plastics. Moreover, we expect this study to influence the research and applications of plastic-degrading fungi to save Earth’s planet.

## 5. Conclusions

Even though plastic is currently an important material in the global environment, it is becoming a huge threat to nature. Because current global plastic production is increasing rapidly (300 million tons annually) and plastics have a very low natural degradation rate, they accumulate in natural environments and cause considerable damage to biodiversity and natural ecosystems. At present, scientists and researchers are assessing the usefulness of microorganisms in accelerating plastic degradation. In this study, we reviewed plastic degradation using fungi. Herein we list more than 200 records of fungi capable of degrading fungi based on the available literature. Their phylogenetic relationships were analyzed using a combined ITS, LSU, SSU, TEF, RPB1, and RPB2 dataset generated from 395 strains. Our results confirm that plastic-degrading fungi are taxonomically diverse and belong to three major fungal phyla—the Ascomycota, Basidiomycota, and Mucoromycota. The Ascomycota plastic degraders belong to five major classes: Dothideomycetes, Eurotiomycetes, Leotiomycetes, Saccharomycetes, and Sordariomycetes. Plastic-degrading Basidiomycota fall within the Agaricomycetes, Microbotryomycetes, Tremellomycetes, Tritirachiomycetes, and Ustilaginomycetes. Mucoromycota fungi capable of degrading plastics were found under the Mucoromycetes. The Eurotiomycetes include the highest number of recorded plastic degraders in the fungi kingdom. However, a wide range of plastic-degrading fungal genera was found within the class Sordariomycetes. Moreover, there is an acute need for future research on similar topics to resolve the global problem of plastic accumulation in nature.

## Figures and Tables

**Table 1:** Annual plastic production [3,9].

Plastic Type and Structure [9]	Main Uses [9]	Annual Production/ Million Metric Tons [35]	Specific Surface Degradation Rate (Min−Max; μm Tear−1) [17]	
			Land	Marine
**Polyethylene Terephthalate (PET or PETE)** [36]	Beverage bottles, Food bottles/jars and polyester clothing or rope	30.5	0	-
**High-Density Polyethylene (HDPE)**	Milk cartons, detergent bottles, cereal box liners, toys, buckets, park benches, and rigid pipes	66.96	1.0 (0.91−1.1)	4.3 (0−11)
**Polyvinyl Chloride (PVC or Vinyl)**	Plumbing pipes, credit cards, human and pet toys, rain gutters, teething rings, IV fluid bags and medical tubing, and oxygen masks	44.3	0	-
**Low-Density Polyethylene (>LDPE) **	Plastic wrap, sandwich and bread bags, bubble wrap, garbage bags, grocery bags and beverage cups	>1500	11	15 (0−37)
**Polypropylene (PP)**	Straws, bottle caps, prescription bottles, hot food containers, packaging tape, disposable diapers, and DVD/CD boxes	56	-	7.5
**Polystyrene (PS or Styrofoam)**	Cups, takeout food containers, shipping and product packaging, egg cartons, cutlery and building insulation	15.61	0	-
**Other**	Eyeglasses, baby and sports bottles, electronics, CD/DVDs, lighting fixtures, and clear plastic cutlery	-	270 (20−1400)	16 (7.5−29)

**Table 2:** Recorded taxa of fungi that are known to degrade plastics.

Fungus	Polymer Hydrolysed	Class	Family	Environment	References
Ascomycota					
*Acremonium kiliense*	PE	Sordariomycetes	Bionectriaceae	Soils	[37]
*Acremonium* sp.	PHB, Poly[3 HB-co-(10 mol%) 3HV]	Sordariomycetes	Bionectriaceae	Soils	[38]
*Alternaria alternata*	PE, LDPE	Dothideomycetes	Pleosporaceae	Dumpsites, Mangrove stands	[19,39]
*Alternaria brassicicola*	-	Dothideomycetes	Pleosporaceae	Not mentioned	[40]
*Alternaria dauci*	PUR	Dothideomycetes	Pleosporaceae	Rainforest	[41]
*Alternaria solani*	PS-PUR	Dothideomycetes	Pleosporaceae	Soils, Wall paints (Latex), Pieces of plastic debris	[42]
*Alternaria* sp.	PUR	Dothideomycetes	Pleosporaceae	Rainforest	[41]
*Arxula adeninivorans*	-	Saccharomycetes	Trichomonascaceae	Not mentioned	[43]
*Aspergillus caespitosus*	LDPE	Eurotiomycetes	Aspergillaceae	Mangrove stands	[19]
*Aspergillus fischeri*	PCL	Eurotiomycetes	Aspergillaceae	Soils	[44,45]
*Aspergillus flavus*	PE, HDPE, LDPE, PVC, PCL, PS-PUR, PEA, PPA, PBA	Eurotiomycetes	Aspergillaceae	Soils	[12,42,44,46,47,48]
*Aspergillus fumigatus*	PHB, Poly[3HB-co-(10 mol%) 3HV], HDPE, LDPE, PS-PUR, Sky-Green, Poly[3HB-co-(7–77 mol%) 3HV], PHV, Poly[3HB-co-(13–61 mol%) 4HB], PES, PEA, PBA, PCL, PBS	Eurotiomycetes	Aspergillaceae	Soils	[12,38,42,49,50,51,52,53,54]
*Aspergillus glaucus*	PE	Eurotiomycetes	Aspergillaceae	Mangrove Soils	[55]
*Aspergillus japonicus*	LDPE	Eurotiomycetes	Aspergillaceae	Polythene polluted sites	[56]
*Aspergillus nidulans*	LDPE	Eurotiomycetes	Aspergillaceae	Dumpsite	[54,57]
*Aspergillus Niger*	PE, HDPE, LDPE, PVC, Sky-Green, PEA, PPA, PBA	Eurotiomycetes	Aspergillaceae	Soils	[12,47,48,50,56,58,59,60,61,62,63,64]
*Aspergillus nomius*	LDPE	Eurotiomycetes	Aspergillaceae	Landfill soils	[65]
*Aspergillus ochraceus*	LDPE	Eurotiomycetes	Aspergillaceae	*Pleurotus ostreatus* (Oyster mushroom) baglog	[63]
*Aspergillus oryzae*	LDPE	Eurotiomycetes	Aspergillaceae	Not mentioned	[54,66,67]
*Aspergillus penicilloides*	PHB	Eurotiomycetes	Aspergillaceae	Biological products	[68]
*Aspergillus* sp.	PE	Eurotiomycetes	Aspergillaceae	Sea water	[69]
*Aspergillus sydowii*	PE	Eurotiomycetes	Aspergillaceae	Dumping sites, Mangrove rhizosphere soils	[2]
*Aspergillus terreus*	LDPE, HDPE, PS-PUR, PE	Eurotiomycetes	Aspergillaceae	Soils	[2,12,19,42,45,49]
*Aspergillus tubingensis*	PU	Eurotiomycetes	Aspergillaceae	Soils	[70]
*Aspergillus ustus*	Sky-Green, PHB	Eurotiomycetes	Aspergillaceae	Soils, Deep Sea	[50,71]
*Aspergillus versicolor*	HDPE, LDPE, PVC, PEA, PPA, PBA	Eurotiomycetes	Aspergillaceae	Soils, Degraded polyimides, Marine water	[47,48,71,72]
*Aureobasidium pullulans*	PCL, PEA, PPA, PBA	Dothideomycetes	Saccotheciaceae	Not mentioned	[48,73]
*Bionectria* sp.	PUR	Sordariomycetes	Bionectriaceae	Rainforest	[41]
*Candida guilliermondii*	PHB	Saccharomycetes	Candidaceae	Deep sea	[71]
*Cephalosporium* sp.	PHB	Sordariomycetes	Incertae sedis	Not mentioned	[74]
*Chaetomium globosum*	HDPE, LDPE, PVC, PCL, PEA, PPA, PBA	Sordariomycetes	Chaetomiaceae	Soils	[44,47,48]
*Chaetomium* sp.	PE	Sordariomycetes	Chaetomiaceae	Groundnut	[46]
*Chrysonilia setophila*	HDPE, LDPE, PVC	Sordariomycetes	Sordariaceae	Soils	[47]
*Cladosporium cladosporioides*	PU	Sordariomycetes	Chaetomiaceae	Plastic debris in a shoreline of a lake	[6]
*Cladosporium* sp.	PHB	Sordariomycetes	Chaetomiaceae	Not mentioned	[74]
*Colletotrichum fructicola*	LDPE	Sordariomycetes	Glomerellaceae	Not mentioned	[64]
*Curvularia lunata*	PE	Dothideomycetes	Pleosporaceae	Dumpsites	[39]
*Curvularia protuberata*	Sky-Green	Dothideomycetes	Pleosporaceae	Soils	[50]
*Debaryomyces hansenii*	PHB	Saccharomycetes	Debaryomycetaceae	Deep sea	[71]
*Diaporthe italiana*	LDPE	Sordariomycetes	Diaporthaceae	Not mentioned	[64]
*Edenia gomezpompae*	PUR	Dothideomycetes	Phaeosphaeriaceae	Rainforest	[41]
*Emericellopsis minima*	PHB, Poly[3HB-co-(30 mol%) 3HV]	Sordariomycetes	Incertae sedis	Not mentioned	[75]
*Eupenicillium hirayamae*	-	Eurotiomycetes	Aspergillaceae	Mangrove stand	[19]
*Eupenicillium rubidurum*	-	Eurotiomycetes	Aspergillaceae	Not mentioned	[45]
*Eupenicillium* sp.	PHB	Eurotiomycetes	Aspergillaceae	Soils	[76]
*Exophiala jeanselmei*	Polyether	Eurotiomycetes	Herpotrichiellaceae	Soils	[77]
*Fusarium moniiforme*	PCL	Sordariomycetes	Nectriaceae	Not mentioned	[78]
*Fusarium oxysporium*	Poly[3HB-co-(12 mol%) 3HV], HDPE, LDPE, PVC, PET	Sordariomycetes	Nectriaceae	Soils	[47,79,80,81]
*Fusarium solani*	LDPE, HDPE, PVC, PCL, PS-PUR, PHB, PET	Sordariomycetes	Nectriaceae	Soils	[12,42,47,49,50,78,81,82,83,84]
*Fusarium* sp.	PE, PCL	Sordariomycetes	Nectriaceae	Soils, Dumpsites	[44,85]
*Gliocladium roseum*	PS-PUR	Sordariomycetes	Hypocreaceae	Not mentioned	[86]
*Gliocladium virens*	LDPE	Sordariomycetes	Hypocreaceae	Not mentioned	[60]
*Glomerella cingulata*	-	Sordariomycetes	Glomerellaceae	Not mentioned	[87,88]
*Guignardia mangiferae*	PUR	Dothideomycetes	Phyllostictaceae	Rainforest	[41]
*Humicola insolens*	-	Sordariomycetes	Chaetomiaceae	Not mentioned	[89]
*Lasiodiplodia* sp.	PUR	Dothideomycetes	Botryosphaeriaceae	Rainforest	[41]
*Leptosphaeria* sp.	PU	Dothideomycetes	Botryosphaeriaceae	Plastic debris in a shoreline of a lake	[6]
*Malbranchea cinnamomea*	-	Eurotiomycetes	Onygenaceae	Not mentioned	[90]
*Monascus* sp.	PU	Eurotiomycetes	Monascaceae	Plastic contaminated soils	[91]
*Monilinia fructicola*	-	Leotiomycetes	Sclerotiniaceae	Not mentioned	[92]
*Myceliophthora thermophila*	-	Sordariomycetes	Chaetomiaceae	Not mentioned	[93]
*Nectria* sp.	PUR	Sordariomycetes	Nectriaceae	Rainforest	[41]
*Paecilomyces farinosus*	Poly[3HB-co-(12 mol%) 3HV], PHB, Sky-Green	Eurotiomycetes	Thermoascaceae	Soils	[50,79]
*Paecilomyces lilacinus*	PHB, PCL, Poly[3HB-co-(12 mol%) 3HV]	Eurotiomycetes	Thermoascaceae	Soils	[79,94]
*Paecilomyces marquandii*	PHB	Eurotiomycetes	Thermoascaceae	Biological products	[67]
*Paecilomyces variotii*	-	Eurotiomycetes	Thermoascaceae	Mangrove stands	[19]
*Paraphoma*-like	-	Dothideomycetes	Phaeosphaeriaceae	Barley phylloplane	[95,96]
*Penicillium adametzii*	PHB	Eurotiomycetes	Aspergillaceae	Biological products	[68]
*Penicillium argillaceum*	PCL	Eurotiomycetes	Aspergillaceae	Not mentioned	[53]
*Penicillium chermisinum*	PHB	Eurotiomycetes	Aspergillaceae	Freshwater	[97]
*Penicillium crysosporium*	Poly[3HB-co-(7 mol%) 3HV]	Eurotiomycetes	Aspergillaceae	Not mentioned	[98]
*Penicillium daleae*	PHB	Eurotiomycetes	Aspergillaceae	Biological products	[68]
*Penicillium dupontii*	PCL	Eurotiomycetes	Aspergillaceae	Not mentioned	[53]
*Penicillium funiculosum*	PCL, PHB, PHV, Poly[3HB-co-(7, 14%) 4HB], Poly[3HB-co-(7, 27, 45, 71%) 3HV], PEA, PPA, PBA	Eurotiomycetes	Aspergillaceae	Soils	[44,48,50,94,99,100]
*Penicillium griseofulvum*	PU	Eurotiomycetes	Aspergillaceae	Plastic debris in a shoreline of a lake	[6]
*Penicillium janthinellum*	PHB	Eurotiomycetes	Aspergillaceae	Freshwater	[97]
*Penicillium minioluteum*	PHB	Eurotiomycetes	Aspergillaceae	Soils	[50]
*Penicillium orchrochloron*	PHB	Eurotiomycetes	Aspergillaceae	Biological products	[68]
*Penicillium oxalicum*	HDPE, LDPE	Eurotiomycetes	Aspergillaceae	Soils of a plastic dumping site	[26]
*Penicillium pinophilium*	PHB, LDPE	Eurotiomycetes	Aspergillaceae	Soils	[50,60,101]
*Penicillium restricum*	PHB	Eurotiomycetes	Aspergillaceae	Biological products	[68]
*Penicillium roqueforti*	PLA	Eurotiomycetes	Aspergillaceae	Not mentioned	[102]
*Penicillium simplicissimum*	PE, PHB, Poly[3HB-co-(7 mol%) 3HV], Sky-Green	Eurotiomycetes	Aspergillaceae	Soils	[50,76,85,97,98,103]
*Penicillium* sp.	PHB, HDPE, LDPE, PVC, PEA, PCL, polyalkylene dicarboxylic acids	Eurotiomycetes	Aspergillaceae	Soils	[47,104,105,106]
*Penicillium verruculosum*	Mater-Bi	Eurotiomycetes	Aspergillaceae	Soils	[50]
*Pestalotiopsis microspora*	PUR	Sordariomycetes	Sporocadaceae	Rainforest	[41]
*Pestalotiopsis* sp.	PUR	Sordariomycetes	Sporocadaceae	Rainforest	[41]
*Phaeosphaeria* sp.	PUR	Dothideomycetes	Phaeosphaeriaceae	Rainforest	[41]
*Phialophora alba*	-	Eurotiomycetes	Herpotrichiellaceae	Mangrove stands	[19]
*Phoma* sp.	HDPE, LDPE, PVC	Dothideomycetes	Didymellaceae	Soils	[45,47]
*Plectosphaerella* sp.	PUR	Sordariomycetes	Plectosphaerellaceae	Rainforest	[41]
*Pleosporales* sp.	PUR	Dothideomycetes		Rainforest	[41]
*Pullularia pullulans*	PEA, PPA, PBA	Dothideomycetes	Saccotheciaceae	Not mentioned	[48]
*Sirococcus conigenus*	-	Sordariomycetes	Gnomoniaceae	Not mentioned	[107]
*Spicaria* spp.	PS-PUR	Incertae sedis	Incertae sedis	Soils, Wall paints (Latex), Plastic debris	[42]
*Stagonosporopsis citrulli*	LDPE	Dothideomycetes	Didymellaceae	Not mentioned	[64]
*Talaromyces islandicus*	-	Eurotiomycetes	Aspergillaceae	Not mentioned	[45]
*Thermoascus aurantiacus*	PHB, PCL, PBS	Eurotiomycetes	Thermoascaceae	Not mentioned	[53]
*Thielavia terrestris*	-	Sordariomycetes	Chaetomiaceae	Soils	[108,109]
*Thyrostroma jaczewskii*	LDPE	Dothideomycetes	Botryosphaeriaceae	Not mentioned	[64]
*Trichoderma hamatum*	LDPE, PS, PVC	Sordariomycetes	Hypocreaceae	Plastic waste material	[110,111]
*Trichoderma reesei*	-	Sordariomycetes	Hypocreaceae	Not mentioned	[112]
*Trichoderma viride*	-	Sordariomycetes	Hypocreaceae	Landfill soils	[65]
*Verticillium Lecanii*	PE	Sordariomycetes	Plectosphaerellaceae	Soils	[37]
*Verticillium leptobactrum*	PHB	Sordariomycetes	Plectosphaerellaceae	Soils	[38]
*Xepiculopsis gramineae*	PU	Sordariomycetes	Incertae sedis	Plastic debris in a shoreline of a lake	[6]
*Zopfiella karachiensis*	PUR	Sordariomycetes	Lasiosphaeriaceae	Rainforest	[41]
Basidiomycota					
*Cryptococcus laurentii*	PCL	Tremellomycetes	Tremellaceae	Soils	[44]
*Cryptococcus magnus*	-	Tremellomycetes	Tremellaceae	Barley Phylloplane	[95]
*Cryptococcus* sp.	PBS, PBSA	Tremellomycetes	Tremellaceae	-	[113]
*Papiliotrema laurentii*	PBS, PBSA	Tremellomycetes	Rhynchogastremaceae	Part of a microbiome analysis of an aircraft	[114]
*Phanerochaete chrysosporium*	LDPE, Poly[3HB-co-(7 mol%) 3HV], PVC	Agaricomycetes	Phanerochaetaceae	Soils	[60,108,115,116,117]
*Pleurotus ostreatus*	PE, LDPE	Agaricomycetes	Pleurotaceae	-	[16]
*Polyporus circinatus*	PHB	Agaricomycetes	Hymenochaetaceae	-	[74]
*Pseudozyma antarctica*	-	Ustilaginomycetes	Ustilaginaceae	Obtained from the culture collection of the Japan Collection of Microorganisms (JCM) of the Riken BioResource Center in Wako, Japan.	[118]
*Rhodosporidium sphaerocarpum*	PHB	Microbotryomycetes	Sporidiobolaceae	Deep sea	[71]
*Tritirachium album*	PLA	Tritirachiomycetes	Tritirachiaceae	-	[119]
Mucoromycota					
*Mucor* sp.	PHB, PVC	Mucoromycetes	Mucoraceae	-	[74,106]
*Mucor hiemalis*	HDPE, LDPE, PVC	Mucoromycetes	Mucoraceae	Soils	[47]
*Rhizopus arrhizus*	PCL, polyalkylene dicarboxylic acids	Mucoromycetes	Rhizopodaceae	-	[120]
*Rhizopus delemar*	PPA, PET copolymers with dicarboxylic acids	Mucoromycetes	Rhizopodaceae	-	[121,122]

PHB: Polyhydroxybutyrate, PUR: Polyurethane, PCL: Polycaprolactone, PPA: Polyphthalamide, PBA: Polybutanamide, PHV: Poly(3-hydroxybutyrate-co-3-hydroxyvalerate), PES: Polyethersulfone, PEA: Polyesteracetals, PBS: Polybutylene succinate, PBSA: Poly(butylene succinate-co-butylene adipate), PET: Polyethylene terephthalate, HDPE: High-density polyethylene, PVC: Polyvinyl chloride, LDPE: Low-density polyethylene, PS-PUR: polyester-polyurethane, PP: Polypropylene, PS: Polystyrene, PE: Polyethylene, PU: Polyurethane. The “-” is used to show the data is not available in literature.

## Data Availability

The original contributions presented in the study are included in the article/Supplementary Material. Further inquiries can be directed to the corresponding authors.

## Supplementary Materials

The following supporting information can be downloaded at: https://www.mdpi.com/article/10.3390/jof8080772/s1, Table S1: Taxa table for phylogenetic analysis.
