# Supplementary material for: A Review of the Fungi That Degrade Plastic
Source: J Fungi (Basel). 2022 Jul 25;8(8):772. doi: 10.3390/jof8080772 (PMC9330918; doi:10.3390/jof8080772)
Supplement: Supplementary file 1 [file jof-08-00772-s001.zip › jof-1762137-supplementary.pdf]

Table S1. Taxa table for phylogenetic analyses.

Taxon Name	Strain	GenBank Accession Number					
		ITS	LSU	SSU	TEF	RPB1	RPB2
Abrothallus buellianus	SPO 303	KF816166	KF816234	KF816217	KF816186	—	—
Acremonium alternatum	CBS 820.70	MH859959	MH871758	—	—	—	—
Acremonium brachypenium	CBS 866.73	MH860815	MH872547	—	—	—	—
Acrospermum adeanum	M 133	EU940180	EU940104	EU940031	—	—	EU940320
Aliquandostipite khaoyaiensis	CBS 118232	—	GU301796	AF201453	GU349048	—	FJ238360
Alternaria alternata	FA-24	KJ947119	—	—	—	—	—
Alternaria alternata	TAA-05	OL673807	—	OL674053	—	—	—
Alternaria brassicicola	AC71	LC440588	—	—	LC480214	—	LC476794
Alternaria brassicicola	CBS 118699	—	KC584259	KC584515	KC584642	—	KC584383
Alternaria dauci	CBS 477.83	MH861626	MH873342	—	KJ718509	—	KJ718334
Alternaria solani	AKoSrF	OK036398	OK036421	OK036375	—	—	—
Anteaglonium parvulum	GKM 1218	—	GQ221880	—	GQ221922	—	—
Aplosporella prunicola	CBS 121167	KF766147	KF766315	KF766229	—	—	—
Arachnomyces minimus	CBS 324.70	—	FJ358274	AJ315167	—	—	—
Arachnomyces nitidus	—	—	AB075351	—	—	—	—
Arthonia coreana	KoLRI 037694	—	KX913670	—	—	—	KX913668
Arthrinium montagnei	AFTOL-ID 951	—	DQ471018	—	DQ842027	DQ842033	DQ842033
Arthroderma curreyi	CBS 138.26	KT155805	AY176726	AJ315165	—	—	—
Ascobolus crenulatus	AFTOL-ID 181	DQ491504	AY544678	AY544721	DQ471061	DQ471132	DQ470893
Ascosphaera apis	CBS 402.96	—	FJ358275	FJ358343	—	FJ358406	—
Ascotaiwania sawadae	SS00051	HQ446340	HQ446363	HQ446283	HQ446306	—	HQ446418
Ascothailandia grenadoidia	SS03615	GQ390282	GQ390267	GQ390252	HQ446309	—	HQ446420
Aspergillus caespitosus	SRRC 308	AY373841	—	—	—	—	—
Aspergillus caespitosus	NRRL 1929	NR_131288	NG_057296	—	—	—	—
Aspergillus fischeri	CBS 544.65	MH858711	MH870352	GU733354	—	—	—
Aspergillus flavus	ATCC 9643	HQ026738	—	—	—	—	—
Aspergillus flavus	FSS63	KC621105	—	—	—	—	—

Aspergillus fumigatus	502.2_10.4	MG970388	—	—	—	—	—
Aspergillus fumigatus	502.3_13.3	MG970369	—	—	—	—	—
Aspergillus glaucus	CBS 297.71	MH860132	MH871905	—	—	—	—
Aspergillus glaucus	CBS 529.65	MH858701	MH870342	—	—	—	—
Aspergillus glaucus	ATCC 16469	—	AY176751	—	—	—	—
Aspergillus japonicus	NRRL 360	EF661216	EF661216	—	—	—	—
Aspergillus nidulans	FGSC A4	KY074657	KY074658	MG779511	—	—	—
Aspergillus nidulans	RIB40	—	—	MG779508	—	—	—
Aspergillus nidulans	Ya10	—	—	MG779507	—	—	—
Aspergillus nidulans	T3	—	—	MG779513	—	—	—
Aspergillus nidulans	MF 109	—	—	MG779504	—	—	—
Aspergillus nidulans	BTK-1	—	—	MG779512	—	—	—
Aspergillus nidulans	AD-Jt-1	—	—	MG779506	—	—	—
Aspergillus nidulans	JAU1	—	—	MG779505	—	—	—
Aspergillus nidulans	AJR1	—	—	MG779510	—	—	—
Aspergillus nidulans	Ya1	—	—	MG779509	—	—	—
Aspergillus niger	F48-02	KX664417	KX664417	—	—	—	—
Aspergillus niger	F21-04	KX664345	KX664345	—	—	—	—
Aspergillus niger	ATCC 9642	FJ195349	—	—	—	—	—
Aspergillus ochraceus	ICMP 939	AF128850	—	—	—	—	—
Aspergillus ochraceus	EN31	FJ427513	—	—	—	—	—
Aspergillus oryzae	RIB40	—	—	MG779508	—	—	—
Aspergillus oryzae	A540	KX462996	—	—	—	—	—
Aspergillus protuberus	AFTOL-ID 5007	—	FJ176897	FJ176842	—	—	FJ238379
Aspergillus sydowii	ASAU-1	KJ524907	KJ524908	—	—	—	—
Aspergillus terreus	—	KP033202	—	—	—	—	—
Aspergillus terreus	IOC_3995	KM116156	—	—	—	—	—
Aspergillus tubingensis	SBRL2	OL444885	—	—	—	—	—
Aspergillus tubingensis	KS-C17	MZ736584	—	—	—	—	—
Aspergillus ustus	CBS 261.67	NR_131284	—	—	—	—	—
Aspergillus ustus	NCC3968	MT994880	—	—	—	—	—
Aspergillus versicolor	S66	KU318417	KU318415	—	—	—	—

Aspergillus versicolor	S61	KU318416	KU318414	—	—	—	—
Aspergillus nomius	NRRL 13137	AF027860	AF027860	—	—	—	—
Aspergillus nomius	NRRL 25393	AF027864	AF027864	—	—	—	—
Aspergillus penicillioides	CBS 540.65	OL772711	OL772711	GU733350	—	—	—
Aureobasidium pullulans	CBS 109810	FJ150901	FJ150953	—	—	—	—
Aureobasidium pullulans	CBS 701.76	FJ150907	FJ150951	—	—	—	—
Aureobasidium pullulans	CBS 298.56	MH857648	MH869192	—	—	—	—
Baeomyces rufus	P78	KJ462264	KJ462341	KR017260	KR017567	—	—
Bagnisiella examinans	CBS 551.66	—	KF766316	GU296139	—	—	GU371746
Basidiobolus ranarum	ATCC 14449	EF392532	EF392421	—	—	—	EF392476
Basidiobolus ranarum	AFTOL-ID 301	AY997030	—	AY635841	DQ282610	—	—
Basidiobolus ranarum	ARSEF 260	EF392519	EF392409	EF392545	—	—	EF392464
Bezerromyces brasiliensis	CBS 141545	KX470390	KX518623	KX518627	KX518631	—	—
Bionectria pityrodes	CBS 102033	AF210672	AF210672	AY249899	—	—	—
Bionectria sesquicillii	CBS 180.88	AF210666	MH873818	—	—	—	—
Bionectria solani	CBS 752.68	AF358246	MH870947	—	—	—	—
Blastobotrys adeninivorans	CBS 8244	EU343811	LR031515	—	—	EU344080	EU343884
Blastobotrys adeninivorans	CBS:7370	KY101742	KY106200	—	—	—	—
Blastobotrys adeninivorans	CBS:7377	KY101745	KY106198	—	—	—	—
Botryosphaeria dothidea	CMW 8000	NR_111146	KF766319	KF766233	—	—	—
Brigantiaea cf. tricolor	Gaya 70	JQ301655	JQ301543	JQ301603	—	JQ301704	JQ301739
Broomella vitalbae	MFLUCC 15-0023	KP757755	KP757751	KP757759	KP757763	—	—
Buergenerula spartinae	ATCC 22848	JX134666	DQ341492	DQ341471	JX134692	JX134720	—
Byssochlamys nivea	CBS 100.11	FJ389934	AY176750	—	—	JN121551	JF417414
Caliciopsis nigra	MA 18191	—	KP144011	—	—	—	—
Caloscypha fulgens	AFTOL-ID 152	DQ491483	DQ247799	DQ247807	DQ471054	DQ471126	DQ247787
Candida guilliermondii	—	AF022719	—	—	—	—	—
Capnodium coffeae	CBS 147.52	AJ244239	DQ247800	DQ247808	DQ471089	—	DQ247788
Capnodium salicinum	—	—	DQ678050	DQ677997	—	—	—
Capronia pilosella	—	DQ826737	DQ823099	DQ823106	DQ840565	DQ840554	DQ840561
Catinella olivacea	UAMH 10679	DQ915483	EF622212	DQ915484	—	—	—
Celothelium cinchonarum	F 17105	—	DQ329020	—	—	—	—

Cenangiopsis quercicola	KL174	LT158425	KX090811	KX090862	KX090663	KX090760	KX090713
Cephalosporium caerulens	KF-140	AY566994	AY567002	AY567008	—	—	—
Cephalosporium gramineum	CBS 132.34	NR_171209	MH866942	HQ322373	—	—	—
Cephalosporium gramineum	MK08302	HQ322344	HQ322372	HQ322372	—	—	—
Chaenotheca brachypoda	Prieto 3023	—	JX000086	—	—	JX000135	—
Chaenotheca furfuracea	Wedin 6366	JX000101	JX000087	JX000068	—	JX000137	—
Chaenotheca trichialis	Prieto 3028	JX000102	JX000085	JX000069	—	JX000136	—
Chaenothecopsis sitchensis	—	JX119102	JX119111	—	—	—	—
Chaetomium globosum	PDL2039	MW522514	—	—	MW530423	—	—
Chaetomium globosum	PDL2040	MW522352	—	—	MW530424	—	—
Chaetomium globosum	CBS 164.62	JN209920	MH869717	—	KF001706	KF001751	KF001796
Chaetomium globosum	CBS 160.62	NR_144851	KT214596	—	KT214704	KT214630	KT214666
Chaetomium longiciliata	LC4055	KP336774	KP336823	—	—	—	KT149497
Chaetomium tectifimeti	CBS 142032	NR_147668	KX976737	—	—	—	KX976836
Chaetothyrium agathis	MFLUCC 12 C0113	KP744437	KP744480	—	—	—	—
Chlorociboria cf. aeruginosa	AFTOL-ID 151	DQ491501	AY544669	AY544713	DQ471053	DQ471125	DQ470886
Chrysothrix candelaris	Frisch 11/Se45	—	KF707640	—	—	—	KF707663
Cladonia caroliniana	AFTOL-ID 3	DQ782832	AY584640	AY584664	DQ782888	DQ782816	AY584684
Cladosporium cladosporioides	CPC 14705	HM148050	—	—	HM148291	—	—
Cladosporium cladosporioides	CPC 15038	HM148051	—	—	HM148292	—	—
Cladosporium cladosporioides	CSPF3	KU508795	—	—	—	—	—
Cladosporium silenes	CBS 109082	NR_111270	JF770463	—	EF679429	—	—
Clonostachys ochroleuca	AFTOL-ID 187	—	DQ862027	DQ862044	DQ862029	—	DQ862013
Colletotrichum gloeosporioides	LC0555	JN943090	JN940412	JN940356	—	JN984972	—
Cordyceps militaris	OSC 93623	JN049825	AY184966	AY184977	DQ522332	DQ522377	—
Corynelia fructigena	AW 250	KP881704	KP881716	KP881720	—	—	—
Cosmospora coccinea	AR2741	—	AY489734	AY489702	AY489629	AY489667	—
Crocynia pyxinoides	AFTOL-ID 111	—	AY584653	AY584677	DQ883767	DQ883735	DQ883748
Cryptococcus aquaticus	CBS 5443	AF410469	AF075470	AB032621	—	—	—
Cryptococcus huempii	CBS 8186	AF444322	AF189844	AB032636	—	—	—
Cryptococcus laurentii	CL19	JN627001	JQ968480	JX393955	—	—	—
Cryptococcus laurentii	CL18	JN627000	JQ968479	JX393954	—	—	—

Cryptococcus macerans	CBS 2206	AF444329	AF189848	AB032642	—	—	—
Cryptococcus magnus	RKI-06-00213	GU979219	GU979218	—	—	—	—
Cryptococcus magnus	CBS140	AF190008	AF181851	—	KF037068	KF036383	KF036796
Cudoniella clavus	AFTOL-ID 166	DQ491502	DQ470944	DQ470992	DQ471056	DQ471128	DQ470888
Curvularia lunata	CATAS-CL33	MW186189	MW186194	—	—	—	—
Curvularia lunata	CATAS-CL26	MW186188	MW186193	—	—	—	—
Curvularia protuberata	CBS 376.65	MH858620	MH870260	—	KM196576	—	HG779176
Cyphellophora laciniata	CBS 190.61	EU035416	FJ358239	FJ358307	—	FJ358370	—
Dactylospora haliotrepha	AFTOL-ID758	—	FJ176855	FJ176802	—	—	FJ238344
Dactylospora mangrovei	AFTOL-ID 2108	—	FJ176890	FJ176836	FJ238411	KJ766849	FJ238375
Dactylospora vrijmoediae	NTOU4002	NR_138396	KC692153	KC692152	—	—	KC692154
Debaryomyces fabryi	CBS:789	KY103198	KY107491	—	—	—	—
Debaryomyces hansenii	SLDY-294	MH782058	—	—	—	—	—
Debaryomyces hansenii	clone 28	—	KF488760	—	—	—	—
Debaryomyces hansenii	clone 187	—	KF488792	—	—	—	—
Debaryomyces maramus	CBS:1958	KY103271	KY107572	—	—	—	—
Diatrype disciformis	AFTOL-ID 927	—	DQ470964	DQ471012	DQ471085	DQ471158	—
Dolabra nepheliae	CBS 122120	—	GU332517	—	GU332523	GU332521	—
Dothidea insculpta	CBS 189.58	AF027764	DQ247802	DQ247810	DQ471081	—	AF107800
Dothiorella iberica	CBS 115041	NR_111165	AY928053	KF766245	—	—	—
Dyfrolomyces tiomanensis	NTOU3636	—	KC692156	KC692155	KC692157	—	—
Edenia gomezpompae	CBS 124106	NR_156217	NG_059202	—	—	—	—
Emericellopsis minima	CBS 388.70	MH859751	MH871510	—	—	—	—
Emericellopsis minima	CBS 488.71	MH860228	MH871996	—	—	—	—
Encephalographa elisae	EB 0347	—	GU397343	GU397358	—	—	—
Encoelia furfuracea	KL107	LT158416	KX090798	KX090850	KX090653	KX090749	KX090701
Endogone pisiformis	KPM:NC0024233	—	LC002630	LC107349	LC107391	LC431135	—
Endogone botryocarpus	KPM NC0026731	—	NG_068251	NG_070795	LC431111	—	—
Epibryon plagiochilae	M187	EU940201	EU940124	—	—	—	EU940337
Eremithallus costaricensis	Luecking 16273	—	EU622919	—	—	EU622921	—
Eurotium herbariorum	NRRL 116	EF652052	U29553	AB002069	—	—	—
Exophiala dermatitidis	—	DQ826738	DQ823100	DQ823107	DQ840566	DQ840555	DQ840562

Exophiala jeanselmei	CBS 507.90	AY156963	KJ930161	—	EF551530	—	—
Exophiala jeanselmei	D12G	MT023611	MT023628	—	—	—	—
Falcocladium thailandicum	CBS 121717	JF831939	JF831934	JF831930	—	KM232286	—
Flammeascoma lignicola	MFLUCC 10-0128	KT324582	KT324583	KT324584	—	—	KT324586
Fusarium solani	NRRL 32492	EU329679	EU329679	—	DQ246990	—	EU329584
Fusarium solani	NRRL 46702	EU329711	EU329711	—	—	—	EU329660
Fusarium solani	MRC 2565	MH582400	—	—	MH582420	—	MH582410
Fusarium solani	NRRL 46699	EU329708	EU329708	—	—	—	EU329657
Fusarium verticillioides	CBS 128816	MH865159	MH876599	—	—	—	—
Fusarium verticillioides	CBS 127178	MH864460	MH875900	—	—	—	—
Fusarium oxysporum	KUMBPJBT-70	OL679453	—	—	—	—	—
Fusarium oxysporum	KUMBNPJBT-69	OL504748	—	—	—	—	—
Geoglossum nigritum	AFTOL_ID 56	DQ491490	AY544650	AY544694	DQ471044	DQ471115	DQ470879
Gibellulopsis nigrescens	DAOM 226890	GU180631	GU180648	GU180613	—	—	GU180664
Gliocladium roseum	G97012	AJ309334	—	—	—	—	—
Glomerella cingulata	ATP0507C	EU358953	EU358953	—	—	—	—
Glomerella cingulata	ATP0407B	EU358952	EU358952	—	—	—	—
Gnomonia gnomon	CBS 199.53	AY818956	AF408361	DQ471019	DQ471094	—	DQ470922
Guignardia mangiferae	1095	EU683671	—	—	EU683652	—	—
Gymnoascus reesii	CBS 259.61	—	FJ358284	FJ358349	—	FJ358411	—
Herpomyces chaetophilus	602b	KT800039	KT800009	KT800023	—	—	—
Herpomyces periplanetae	602c	KT800041	KT800010	KT800025	—	—	—
Humicola insolens	CBS 147.64	MH858398	MH870026	—	—	—	—
Hysterium angustatum	CBS 236.34	—	FJ161180	GU397359	FJ161096	GU456341	FJ161117
Kylindria peruamazonensis	CBS 421.95	GU291800	HM237325	—	—	—	—
Laboulbenia pedicellata	H84-1	—	KY350537	KY523244	—	—	—
Lecanographa amylacea	Thor 26176	—	KF707639	—	—	—	KF707659
Leptosphaeria sp.	KK-2015	KP747710	—	—	—	—	—
Leptosphaeria irregularis	MFLUCC 15-1118	NR_171725	KX856055	—	KX856058	—	KX856057
Letrouitia domingensis	Gaya 55	JQ301673	JQ301569	JQ301625	—	JQ301719	JQ301764
Letrouitia parabola	Gaya 11	JQ301675	JQ301570	JQ301626	—	JQ301720	JQ301765
Lichenoconium aeruginosum	JL359-09	—	HQ174269	—	—	—	—

Lichenoconium erodens	JL363-09	—	HQ174267	—	—	—	—
Lichenothelia arida	L2198	—	KR045755	—	—	—	—
Lichinella iodopulchra	AFTOL-ID 896	DQ842016	DQ782916	—	DQ832327	DQ782857	DQ832328
Lindra thalassiae	AFTOL-ID 413	DQ491508	DQ470947	DQ470994	DQ471065	DQ471139	DQ470897
Lipomyces chichibuensis	NBRC 109582	AB828723	AB828721	—	—	—	—
Lipomyces spencermartinsiae	CBS 5608	JN943148	NG_055385	—	DQ496132	—	JN985491
Loramyces macrosporus	AFTOL-ID 913	JN033383	DQ470957	DQ471005	DQ471076	DQ471149	DQ470907
Loxosporopsis corallifera	T1087	KR017089	KR017219	KR017264	KR017572	—	KR017516
Macrohilum eucalypti	CPC 10945	DQ195781	DQ195793	DQ195804	—	—	—
Malbranchea cinnamomea	CBS 343.55	MH857506	MH869046	JQ067912	—	—	—
Malbranchea cinnamomea	CBS 960.72	JF922020	—	GU733363	—	—	—
Manglicola guatemalensis	BCC 20156	JN819283	FJ743448	FJ743442	—	—	—
Marcelleina persoonii	AFTOL-ID 164	—	DQ470943	DQ470991	DQ471055	DQ471127	DQ470887
Melanomma pulvis-pyrius	CBS 124080	—	GU456323	GU456302	GU456265	—	GU456350
Melanops tulasnei	CBS 116805	NR_138372	KF766365	KF766282	—	—	—
Metschnikowia noctiluminum	CBS:9907	GU931705	KY108488	—	KC859829	—	KC859706
Microascus trigonosporus	AFTOL-ID 914	DQ491513	DQ470958	DQ471006	DQ471077	DQ471150	DQ470908
Microbotryum violaceum	GLM 50283	NR_119596	NG_042441	—	—	—	—
Microbotryum adenopetalae	KRAM F 55201	NR_119576	NG_042418	—	—	—	—
Microbotryum silenes-saxifragae	KR M 23890	NR_119964	NG_042585	—	—	—	—
Mollisia cinerea	AFTOL-ID 76	DQ491498	DQ470942	DQ470990	DQ471051	DQ471122	DQ470883
Monascus ruber	ATCC 16371	AY498572	AF364996	—	—	—	—
Monascus ruber	PUR_73	KX495607	—	—	—	—	—
Monascus sanguineus	PUR_193	KX495608	—	—	—	—	—
Monascus sp.	PUR_196	KX495609	—	—	—	—	—
Monilinia fructicola	M126	KY781235	—	—	—	—	—
Monilinia fructicola	M127	KY781236	—	—	—	—	—
Monilochaetes dimorphospora	MUCL 40959	NR_137765	HQ609480	HQ609487	—	—	—
Morchella importuna	HKAS 55009	**MG871294**	**MG871328**	**MG859242**	**MG980694**	—	**MG980719**
Mortierella cf. wolfii	CBS 614.70	JX975975	HQ667420	HQ667500	—	—	—
Mortierella wolfii	CBS 209.69	JN943805	JN940864	JQ040256	—	JN985290	—
Mucor ardhlaengiktus	CBS 210.80	JN206172	MH873025	MT523946	—	—	—

Mucor hiemalis	CBS 127123	MH864430	MH875868	—	—	—	—
Mucor hiemalis	CBS 126912	MH864336	MH875781	—	—	—	—
Mucor zychae	CBS 389.35	MH855717	MH867229	—	—	—	—
Mucor janssenii	CBS 185.68	JN206006	MH870818	MT523921	—	—	—
Myceliophthora thermophila	UAMH 2474	HQ724321	HQ724320	—	—	—	—
Mycocalicium subtile large	Tibell 16744	—	AY796004	—	—	—	—
Myriangium hispanicum	CBS 247.33	KX887304	GU301854	GU296180	GU349055	GU357775	GU371744
Myrothecium gramineum	CY176	HQ608010	—	—	—	—	—
Mytilinidion mytilinellum	CBS 303.34	—	FJ161184	FJ161144	FJ161100	GU357810	FJ161119
Nectria cinnabarina	GJS 89-107	—	U00748	—	AF543785	—	—
Nectria mariae	A.R. 4274	JF832629	JF832684	—	JF832542	JF832789	—
Nectria mariae	CBS 125294	NR_160238	—	—	JF832542	—	KM232404
Neolecta irregularis	AFTOL-ID 1363	—	DQ470986	—	—	—	—
Neolecta vitellina	AFTOL-ID 1362	—	DQ470985	—	—	—	—
Neophaeococcomyces aloes	CPC 21873	KF777182	KF777234	—	—	—	—
Neurospora sitophila	—	GU192459	—	—	—	—	—
Niesslia exilis	CBS 357.70	—	AY489718	AY489686	AY489613	AY489645	—
Olpidium brassicae	AFTOL-ID 633	NR_119544	—	DQ322624	DQ275349	—	—
Onygena equina	ATCC 22731	—	AY176717	—	—	—	—
Ophiocordyceps aurantiaca	OSC 128578	JN049833	DQ518770	DQ522556	DQ522345	DQ522391	DQ522445
Ophiocordyceps gracilisOSC	OSC 151906	—	KJ878890	KJ878923	KJ878969	—	—
Ophiodiaporthe cyatheae	YMJ 1364	JX570889	JX570891	JX570890	KC465406	—	JX570893
Ophiostoma piliferum	AFTOL-ID 910	—	DQ470955	DQ471003	DQ471074	DQ471147	—
Orbilia auricolor	AFTOL-ID 906	DQ491512	DQ470953	DQ471001	DQ471072	—	DQ470903
Orbilia vinosa	AFTOL-ID 905	DQ491511	DQ470952	DQ471000	DQ471071	DQ471145	—
Oxydothis metroxylonis	MFLUCC 15-0806	KY206776	KY206765	KY206771	KY206780	—	KY206782
Paecilomyces brunneolus	CBS 370.70	EU037050	—	—	—	—	—
Paecilomyces farinosus	MYA 3388	DQ374646	—	—	—	—	—
Paecilomyces farinosus	RCEF446	AF368797	—	—	—	—	—
Paecilomyces lilacinus	IFM 53352	AB363751	—	—	—	—	—
Paecilomyces lilacinus	AW2-1121	AB558287	—	—	—	—	—
Paecilomyces marquandii	CBS 182.27	AY624193	EF468845	EF468990	—	—	—

Papulosa amerospora	AFTOL-ID 748	—	DQ470950	DQ470998	DQ471069	DQ471143	DQ470901
Paraphoma sp.	B47-9	AB693768	LC126021	LC126020	—	—	—
Paraphoma radicina	CBS 111.79	MH861183	MH872952	EU754092	KF253130	—	KF252180
Parengyodontium album	CBS 368.72	MH860502	MH872217	—	LC382183	—	—
Parengyodontium album	CBS 836.71	MH860372	MH872118	—	LC382178	—	—
Patellaria atrata	CBS 958.97	—	GU301855	GU296181	GU349038	GU357749	GU371726
Peltula euploca	Buedel 14921c-1	MF766354	MF766395	MF766272	—	—	—
Peltula hassei	Buedel 14354a	MF766365	MF766406	MF766283	—	—	—
Penicillium adametzii	CBS 130200	MH865748	MH877181	—	—	—	—
Penicillium chermesinum	CBS 231.81	MH861332	MH873092	—	—	—	MN969111
Penicillium daleae	CBS 114839	MH862971	NG_064184	—	—	—	—
Penicillium funiculosum	KCTC16072	—	—	AF245267	—	—	—
Penicillium funiculosum	NRRL 6417	GQ221866	GQ221866	—	—	—	—
Penicillium funiculosum	NRRL 35431	GU183120	GU183120	—	—	—	—
Penicillium griseofulvum	PG3	KJ467353	—	—	—	—	—
Penicillium hirayamae	CBS 238.65	MH858553	—	—	JN626135	—	—
Penicillium janthinellum	CBS 129616	MH865455	MH876915	—	—	—	—
Penicillium minioluteum	CBS 642.68	AY213674	AY213618	—	—	—	JF417443
Penicillium oxalicum	MSS1026	MZ157156	—	—	—	—	MZ190931
Penicillium pinophilum	NRRL 25074	GQ428200	GQ428200	—	—	—	—
Penicillium pinophilum	NRRL 58691	GQ337428	GQ337428	—	—	—	—
Penicillium pinophilum	NRRL 6420	GQ221867	GQ221867	—	—	—	—
Penicillium roqueforti	CCDM 294	MW600470	—	—	—	—	—
Penicillium roqueforti	CCDM 296	MW600465	—	—	—	—	—
Penicillium simplicissimum	CBS 281.58	MH857784	MH869319	—	—	—	—
Penicillium simplicissimum	CBS 340.48	MH856376	MH867923	—	—	—	—
Penicillium simplicissimum	CBS 280.39	MH856014	MH867512	—	—	—	—
Penicillium verruculosum	SG3	KC416630	JX863916	—	—	—	—
Penicillium verruculosum	ATHUM 5186	FJ004320	FJ004378	—	—	—	FJ004498
Pestalotiopsis microspora	E2520A	KT996035	—	—	—	—	—
Pestalotiopsis microspora	E2708A	KT996045	—	—	—	—	—
Pestalotiopsis microspora	E2711A	KT996047	—	—	—	—	—

Pestalotiopsis microspora	E2712A	KT996048	—	—	—	—	—
Pestalotiopsis microspora	E2911H	KT996057	—	—	—	—	—
Pestalotiopsis microspora	E3412F	KT996065	—	—	—	—	—
Phaeosphaeria sinensis	MFLUCC 18-1552	NR_163350	NG_070076	NG_065788	MK360072	—	—
Phaeosphaeria breonardiae	CPC 25944	NR_155675	KX228345	—	KX228379	—	—
Phaffomyces thermotolerans	CBS:7012	KY104502	KY108772	—	—	—	—
Phanerochaete australosanguinea	MA Fungi 91309	NR_164585	NG_066411	—	—	—	—
Phanerochaete chrysosporium	CBS 481.73	MH860750	MH872462	—	—	—	—
Phanerochaete chrysosporium	CBS 363.65	MH858610	MH870252	—	—	—	—
Phanerochaete concrescens	Spirin 7322	NR_155027	KP994382	—	—	—	—
Phanerochaete tamariciphila	CBS 339.95	MH862517	MH874157	—	—	—	—
Phialophora alba	ICMP:17034	HM116755	—	—	—	—	—
Phoma odoratissimi	CGMCC 3.17488	KP330435	KP330447	—	—	—	KP330406
Phoma herbarum	CBS 615.75	KF251212	KF251715	EU754087	KF253168	—	KF252217
Pichia manshurica	CBS:209	KY104597	KY108860	—	—	—	—
Plectosphaerella populi	CBS 139624	KR476751	MH878144	—	—	—	—
Pleostigma jungermannicola	M174	EU940195	EU940119	EU940046	—	—	EU940331
Pleurotheciella rivularia	CBS 125238	NR_111711	JQ429232	JQ429244	—	—	JQ429263
Pleurotus cystidiosus	CBS 100129	NR_103594	NG_057793	—	—	—	—
Pleurotus ostreatus	PLO6	KC782771	—	—	—	—	—
Pleurotus ostreatus	CCRC 36524	AY265830	—	—	—	—	—
Polyblastia viridescens	AFTOL-ID 2240	—	EF643771	EF689855	—	EF689774	—
Polyporus arculatius	Cui 11398	KU189766	KU189797	KU189826	KU189911	KU189884	KU189980
Polyporus brumalis	Cui 10750	KU189765	KU189796	KU189825	KU189910	KU189883	KU189979
Polyporus ciliatus	Wei 1582	KU189767	KU189798	KU189827	KU189912	—	KU189981
Polyporus squamosus	Cui 10595	KU189778	KU189809	KU189840	KU189925	KU189892	KU189988
Polyporus umbellatus	Pen 13513	KU189772	KU189803	KU189832	KU189917	KU189887	KU189985
Polyporus vatius	Cui 12249	KU507581	KU507583	KU507587	KU507591	KU507589	KU507592
Polyporus vatius	Dai 13874	KU189777	KU189808	KU189838	KU189923	KU189891	KU189987
Polytolypa hystricis	UAMH 7299	AY527405	AY176718	—	—	—	—
Pseudohalonectria hampshirensis	MFLUCC 15-0774	—	KX426218	KX426221	KX426224	—	—
Pseudotulostoma japonica	TNS-F11152	—	AB161194	—	—	—	—

Pseudozyma abaconensis	CBS8380	FJ008053	FJ008047	—	—	—	—
Pseudozyma alboarmeniaca	DMST17135	AB117961	AB117961	—	—	—	—
Pseudozyma antarctica	JCM10317	AB089358	AB089359	—	—	—	—
Pseudozyma antarctica	JCM3941	AB089360	AB089361	—	—	—	—
Pseudozyma brasiliensis	GHG001	KF737866	KF737866	KF737866	—	—	—
Pulvinula archeri	BAP 458	—	DQ220392	U62012	KC109270	JX943662	JX943771
Pyrenula cruenta	—	KC592268	AF279407	AF279406	—	—	AY641067
Pyrgillus javanicus	—	DQ826741	DQ823103	DQ823110	—	—	DQ842009
Pyronema domesticum	AFTOL-ID 949	DQ491517	NG_027655	NG_013185	DQ471093	DQ471166	DQ247795
Pyxidiophora arvernensis	AFTOL-ID 2197	—	FJ176894	FJ176839	FJ238412	—	FJ238377
Rasamsonia argillacea	CBS 408.73	MH860718	MH872429	—	—	—	—
Rasamsonia argillacea	CBS 128787	JF417475	—	—	—	—	—
Rhizina undulata	AFTOL-ID 918	—	DQ470961	DQ471009	DQ471080	DQ471153	DQ470911
Rhizopus americanus	CBS 340.62	HM999967	NG_057873	NG_062623	AB512262	—	—
Rhizopus arrhizus	IAL 7305	OL584187	—	—	—	—	—
Rhizopus arrhizus	CBS 112.07	NR_103595	NG_056282	NG_062621	AB281528	KJ566313	—
Rhizopus delemar	CBS 392.95	MH862535	MH874170	—	—	—	—
Rhizopus delemar	CBS 406.51	MH856921	MH868444	—	AB281555	KJ566366	—
Rhizopus oryzae	NBRC 4759	AB512275	—	—	AB512272	—	—
Rhodosporidium sphaerocarpum	CBS 9082	AF444659	AF444754	—	—	—	—
Rhodosporidium sphaerocarpum	CBS 9079	AF444656	AF444751	—	—	—	—
Rhodosporidium babjevae	UCDFST 04-877	KR149271	KU609429	—	—	—	—
Rhodosporidium diobovatum	TEMD15	KC182127	JQ779973	—	—	—	—
Rhytidhysteron rufulum	MFlucc 14-0577	Ku377560	Ku377565	Ku377570	Ku510399	—	—
Roccella montagnei	Ertz 13069	GU137908	GU138026	—	—	—	GU137662
Roccellographa cretacea	AFTOL-ID 93	—	DQ883696	DQ883705	DQ883733	DQ883716	DQ883713
Saccharomyces cerevisiae	CBS:457	KY105081	KY109267	—	—	—	—
Saccharomycodes ludwigii	CBS:2624	KY105245	KY109478	—	—	—	—
Saccharomycopsis guyanensis	CLIB 1454	HG939420	HG764731	—	—	—	—
Sarcinomyces petricola	—	AJ244274	FJ176893	Y18702	—	—	FJ238376
Sarocladium kiliense	CBS 122.29	AJ621775	HQ232052	HQ232198	—	—	—
Sarocladium kiliense	CBS 157.61	MH858006	MH869564	—	—	—	—

Savoryella lignicola	NF00204	HQ446357	HQ446378	HQ446300	HQ446334	—	—
Sirococcus conigenus	CBS 119615	NR_145277	—	—	EF512532	—	—
Sphaerocreas pubescens	NBRC 109377	—	LC107618	—	LC107619	LC431150	—
Sphaerostilbella berkeleyanan	CBS 102308	KU382208	U00756	AF543770	AF543783	AY489671	DQ522465
Sphinctrina leucopoda	Kalb 33829	AY795875	AY796006	—	—	—	—
Spiromastix warcupii	—	DQ782848	DQ782909	DQ782882	—	—	DQ782870
Stachybotrys chlorohalonata	UAMH6417	AF206273	AY489712	AY489680	AY489607	AY489640	—
Stephanonectria keithii	GJS92-133	—	AY489727	AY489695	AY489622	AY489657	—
Symbiotaphrina buchneri	CBS:6902	—	KY109806	—	—	—	—
Symbiotaphrina microtheca	DVLH	—	KJ004453	—	—	—	—
Taphrina deformans	AFTOL-ID 1234	—	DQ470973	DQ471024	DQ471097	DQ471170	DQ470927
Taphrina wiesneri	IAM 14515	—	AY548292	AY548293	—	—	AY548298
Thermoascus aurantiacus	CBS 398.64	MH858464	MH870100	—	—	—	—
Thermoascus aurantiacus	CBS 415.62	MH858196	MH869794	—	—	—	—
Thermoascus crustaceus	CBS 374.62	—	FJ358289	—	—	—	—
Thermomyces dupontii	CBS 236.58	MH857768	MH869301	MT365146	—	—	JN121611
Thielavia terrestris	LPHT 226	MH305267	—	—	—	—	—
Thyridium vestitum	AFTOL ID 172	—	AY544671	AY544715	DQ471058	DQ471129	DQ470890
Torpedospora radiata	AFTOL-ID 751	—	DQ470951	DQ470999	DQ471070	DQ471144	DQ470902
Tremella austral	Wu 154	MT445848	MT425188	—	MT445760	—	MT445753
Tremella brasiliensis	CBS 6966R	AF444429	AF189864	KF036694	KF037200	–	KF036938
Tremella flava	CBS 8471R	KY105681	KY109891	KF036699	KF037205	KF036527	KF036943
Tremella fuciformis	CBS 6970R	KY105683	AF075476	KF036701	KF037207	KF036529	—
Trichaleurina javanica	HKAS 88981	**MG871291**	**MG871326**	**MG859241**	**MG980693**	—	**MG980716**
Trichoderma harzianum	CECT 2413	AF278790	—	—	—	—	—
Trichoderma reesei	T1	MZ650922	—	—	—	—	—
Trichoderma virens	CBS 127064	MH864402	MH875844	—	—	—	—
Trichoderma viride	CBS 101526	AY380908	—	—	AY376053	—	EU248599
Trichoglossum hirsutum	AFTOL_ID 64	DQ491494	AY544653	AY544697	DQ471049	DQ471119	DQ470881
Trichomerium deniqulatum	MFLUCC10-0884	JX313654	JX313660	—	—	—	—
Trichomonascus apis	CBS:10923	KY105700	KY109911	—	—	—	—
Trichomonascus ciferrii	NBRC 1854	LC158133	LC158142	—	—	—	—

Trigonopsis californica	CBS:10351	KY105760	KY109968	—	—	—	—
Trigonopsis variabilis	UCDFST:75-19	KY037861	KY037829	—	—	—	—
Tritirachium cinnamomeum	CBS 182.42	NR_145372	NG_063987	NG_064977	—	—	—
Tritirachium oryzae	CBS 324.81	MH861349	MH873108	—	—	—	—
Tritirachium roseum	CBS 183.42	NR_155894	KF258731	NG_062824	—	—	JF779656
Umbelopsis ramanniana	CGMCC 3.6646	MF417288	MF417113	MF417068	—	—	—
Ustilago esculenta	MAFF305619	AB211929	AB211929	—	—	—	—
Ustilago nunavutica	DAOM 91211	KF381025	KF381049	—	—	—	—
Valsaria lopadostomoides	CBS 139062	NR_137968	KP687868	KP687972	KP688037	—	KP687943
Verrucaria rupestris	SS043	EU553501	EU598724	—	—	EU723786	—
Verticillium lecanii	IMI 304807	AJ292382	—	—	—	—	—
Verticillium leptobactrum	UFMGCB 6292	KC811000	—	—	—	—	—
Xenocylindrosporium kirstenboschense	—	GU229890	GU229891	—	—	—	—
Xylobotryum portentosum	—	MH468792	MH468792	MH468792	—	MH468799	MH468795
Xylobotryum portentosum	—	MH468791	MH468791	MH468791	—	MH468798	MH468794
Xylona heveae	TC269	JQ838225	JQ838239	JQ838236	—	JQ838243	JQ838246
Xylona heveae	TC161	JQ838232	JQ838238	JQ838237	—	JQ838242	JQ838244
Zopfiella karachiensis	CBS 657.74	MH872621	KP981447	—	—	—	KP981630
